# *Ptpn22* and *Cd2* Variations Are Associated with Altered Protein Expression and Susceptibility to Type 1 Diabetes in Nonobese Diabetic Mice

**DOI:** 10.4049/jimmunol.1402654

**Published:** 2015-10-05

**Authors:** Heather I. Fraser, Sarah Howlett, Jan Clark, Daniel B. Rainbow, Stephanie M. Stanford, Dennis J. Wu, Yi-Wen Hsieh, Christian J. Maine, Mikkel Christensen, Vijay Kuchroo, Linda A. Sherman, Patricia L. Podolin, John A. Todd, Charles A. Steward, Laurence B. Peterson, Nunzio Bottini, Linda S. Wicker

**Affiliations:** * Juvenile Diabetes Research Foundation/Wellcome Trust Diabetes and Inflammation Laboratory, Department of Medical Genetics, Cambridge Institute for Medical Research, University of Cambridge, Cambridge CB2 0XY, United Kingdom;; † Division of Cell Biology, La Jolla Institute for Allergy and Immunology, La Jolla, CA 92037;; ‡ La Jolla Institute for Allergy and Immunology, Type 1 Diabetes Research Center, La Jolla, CA 92037;; § Department of Immunology and Microbial Sciences, The Scripps Research Institute, La Jolla, CA 92037;; ¶ Center for Neurologic Diseases, Brigham and Women’s Hospital and Harvard Medical School, Boston, MA 02115;; ‖ Department of Pharmacology, Merck Research Laboratories, Rahway, NJ 07065; and; # The Wellcome Trust Sanger Institute, Wellcome Trust Genome Campus, Hinxton CB10 1HH, United Kingdom

## Abstract

By congenic strain mapping using autoimmune NOD.C57BL/6J congenic mice, we demonstrated previously that the type 1 diabetes (T1D) protection associated with the *insulin-dependent diabetes* (*Idd*)*10* locus on chromosome 3, originally identified by linkage analysis, was in fact due to three closely linked *Idd* loci: *Idd10*, *Idd18.1*, and *Idd18.3*. In this study, we define two additional *Idd* loci—*Idd18.2* and *Idd18.4*—within the boundaries of this cluster of disease-associated genes. *Idd18.2* is 1.31 Mb and contains 18 genes, including *Ptpn22*, which encodes a phosphatase that negatively regulates T and B cell signaling. The human ortholog of *Ptpn22*, *PTPN22*, is associated with numerous autoimmune diseases, including T1D. We, therefore, assessed *Ptpn22* as a candidate for *Idd18.2*; resequencing of the NOD *Ptpn22* allele revealed 183 single nucleotide polymorphisms with the C57BL/6J (B6) allele—6 exonic and 177 intronic. Functional studies showed higher expression of full-length *Ptpn22* RNA and protein, and decreased TCR signaling in congenic strains with B6-derived *Idd18.2* susceptibility alleles. The 953-kb *Idd18.4* locus contains eight genes, including the candidate *Cd2*. The CD2 pathway is associated with the human autoimmune disease, multiple sclerosis, and mice with NOD-derived susceptibility alleles at *Idd18.4* have lower CD2 expression on B cells. Furthermore, we observed that susceptibility alleles at *Idd18.2* can mask the protection provided by *Idd10/Cd101* or *Idd18.1/Vav3* and *Idd18.3.* In summary, we describe two new T1D loci, *Idd18.2* and *Idd18.4*, candidate genes within each region, and demonstrate the complex nature of genetic interactions underlying the development of T1D in the NOD mouse model.

## Introduction

The NOD mouse spontaneously develops autoimmune diabetes and other endocrine autoimmune disorders (1), and has greatly facilitated studies of type 1 diabetes (T1D) (2). To identify genes associated with T1D, our laboratory uses a congenic strain mapping strategy. Through the construction of a series of congenic strains, by the introgression of segments of DNA from the T1D-resistant mouse strains B6 or C57BL/10J (B10) onto the genetic background of the susceptible NOD mouse strain, we have previously located 14 *insulin-dependent diabetes (Idd)* loci (3). Five B6 protective loci—in which B6-derived alleles, compared with NOD-derived alleles, confer T1D protection—have been identified on chromosome 3: *Idd3*, *Idd17*, *Idd10*, *Idd18.3*, and *Idd18.1* (4–7). In addition to T1D protection, B6- or B10-derived alleles, compared with NOD-derived alleles, can also confer T1D susceptibility. *Idd7* and *Idd8* loci, identified with linkage analysis, have B10-derived alleles that confer susceptibility to T1D (8), and congenic strain mapping has established *Idd14* as a B6-susceptibility allele (9) and *Idd5.4* as a B10-susceptibility allele (10).

In this study, we report the identification of an additional B6-susceptibility allele between *Idd10* and *Idd18.3*, designated *Idd18.2*. The 1.31-Mb *Idd18.2* locus contains 18 protein-encoding genes, including two strong candidates based on their known immune functions, *Ptpn22* and *Nras*. The human ortholog of *Ptpn22*, *PTPN22*, is associated with T1D and 15 other autoimmune diseases, including rheumatoid arthritis, Graves disease, Addison disease, and systemic lupus erythematosus (11). In addition, a functional coding variant of rat *Ptpn22* has been shown to be associated with T1D in the BioBreeding rat (12). *PTPN22* encodes a hematopoietic cell lineage-specific protein tyrosine phosphatase (LYP and PEP in human and mouse, respectively) that negatively regulates T (13) and B cell signaling (14, 15). We identified 183 single nucleotide polymorphisms (SNPs), 6 exonic and 177 intronic, between the B6- and NOD-derived alleles of *Ptpn22* suggesting a likely functional difference. Further investigation revealed that spleen cells and thymocytes from mice with B6-derived susceptibility alleles at *Idd18.2* express higher levels of *Ptpn22* mRNA and PEP protein than the equivalent cell populations from NOD mice.

The congenic strain mapping strategy used to localize *Idd18.2* also revealed an additional *Idd* locus, *Idd18.4*, which is 953 kb long and is immediately telomeric to *Idd10*. *Idd18.4* contains eight genes, including the candidate *Cd2*. CD2 is a cell adhesion molecule expressed on immune cells. The primary ligand of CD2 is CD58 in humans and CD48 in rodents (16), and the interactions between these molecules is important in the formation of the immunologic synapse (17). The CD2/CD58/CD48 pathway is associated with the human autoimmune diseases multiple sclerosis and rheumatoid arthritis (18, 19) and with a model of murine lupus (20). Our studies reveal that congenic strains with NOD-derived susceptibility alleles at *Idd18.4* have lower CD2 expression on B cells.

## Materials and Methods

### Oligonucleotides and genotyping

Primer3 (21) was used to design primers for PCR, RT-PCR, and primer and probe sets for real-time quantitative RT-PCR (qPCR). These were synthesized by Sigma-Genosys; the probes were dual labeled with TAMRA and FAM fluorescent dyes. Sequences of D3Nds and D3Mit microsatellite markers are available at http://www-gene.cimr.cam.ac.uk/todd/public_data/mouse/NDS/NDSMicrosTop.html and http://www.ensembl.org/Mus_musculus/Info/Index, respectively. Other Idd10 and Idd18 markers have been published previously (7, 22). All remaining primers and probes used in this study are available in Supplemental Tables I, IIB, and IID. Methods for DNA extraction, microsatellite, and RFLP genotyping have been described previously (7).

### Animals and diabetes frequency studies

All mice were housed under specific pathogen-free conditions, and the appropriate institutional review committee approved experimental procedures. NOD/MrkTacfBR (henceforth designated as NOD) mice were purchased from Taconic Farms. The derivation of the following congenic strains has been described previously: line 3538, NOD.B6 *Idd10* (N16) (22); line 1538, NOD.B6 *Idd3 Idd10 Idd18*^R323^ (N12) (4); line 1100, NOD.B6 *Idd3 Idd10*^R20^ (N12) (7); and line 1101, NOD.B6 *Idd10*^R2^ (N8) (5), now designated as line 7754 (N9) (7). R8 (N9) was developed contemporaneously with the strains described in Podolin et al. (5).

To develop line 2410 (NOD.B6 *Idd10*, *Idd18.3*, *Idd18.1*; N14), mice from line 1100 were crossed to line 1538, and the resulting progeny were intercrossed. F2 progeny with a recombination event immediately centromeric to *Idd18.3*, resulting in a chromosome that contained B6-derived alleles at *Idd10* (from line 1100) and B6-derived alleles at *Idd18.3* and *Idd18.1* (from line 1538), but NOD-derived alleles between *Idd10* and *Idd18.3* were backcrossed to NOD. Progeny heterozygous for the desired recombinant chromosome were backcrossed to NOD again to remove the B6-derived alleles at *Idd3* by recombination. The resultant progeny were bred to homozygosity. To develop line 3539 (NOD.B6 *Idd18.3*, *Idd18.1*; N16), line 2410 was crossed to NOD and progeny were intercrossed; F2 progeny with a single B6-derived allele at *Idd18* were selected, backcrossed to NOD, and bred to homozygosity. To develop lines 7848 (NOD.B6 *Idd18.4*, *Idd18.2*; N11) and 8010 (NOD.B6 *Idd18.4*; N11) B6 mice were crossed to NOD mice, and progeny were backcrossed again to NOD for several generations while selecting for recombinants as close as possible to *Ptpn22.* The recombinant leading to line 7848 had a B6 allele spanning *Cd2* and *Ptpn22*, and was backcrossed to NOD, intercrossed, and bred to homozygosity. During this intercrossing, an additional recombinant was found that had retained the B6 allele at *Cd2* but had lost the allele at *Ptpn22*. This recombinant was backcrossed to NOD and bred to homozygosity to develop line 8010. Cumulative diabetes frequency studies were conducted and analyzed as described previously (7).

### Verification of Ptpn22 and Cd2 gene structure, resequencing of Ptpn22 in the NOD mouse strain, and identification of *Ptpn22* and *Cd2* polymorphisms

To verify the *Ptpn*22 genetic structure, the mRNA sequence M90388 was aligned to the B6 bacterial artificial chromosome (BAC) clone spanning *Ptpn22,* AC124698, using est2genome (23). The genetic structure of *Cd2* was verified in the same manner with mRNA sequence Y00023 and B6 BAC clone AC131184. *Cd2* SNPs and additional polymorphisms were identified by manually comparing the genomic sequence spanning 2.5 kb upstream of the initiation codon to 2.5 kb downstream of the polyadenylation signal (total distance, 16,945 bp) using AC131184 and the NOD BAC clone, AL672260.25.

To resequence *Ptpn22* in the NOD mouse strain, the BAC clone end sequences from the NOD library were aligned against the B6 mouse genome sequence (24). From this, three NOD BAC clones spanning *Ptpn22*, forming a 465-kb minimal sequencing tile path, were sequenced at the Wellcome Trust Sanger Institute (WTSI) and deposited at the European Molecular Biology Laboratory (http://www.ebi.ac.uk; clone DN-252N9, accession number CU210935; DN-31A8, CU210953; and DN-69P21, CU210868). To identify *Ptpn22* and *Cd2* SNPs computationally between NOD and B6, the NOD BAC clone sequences were fragmented into 1-kb sequences and aligned to the B6 mouse chromosome 3 sequence (NCBIM37) using the sequence search and alignment by hashing algorithm program (25); detected SNPs were filtered using RepeatMasker (26) to exclude SNPs present in regions of repeats or low complexity. The alignments spanning the coding sequence and splice sites of *Ptpn22* were checked manually to confirm SNPs and to identify additional polymorphisms.

Sequence from whole genomic next-generation sequencing (NGS) for the related NOD/ShiLtJ strain (25.3-fold coverage) has become available through the Mouse Genomes Project at the WTSI (http://www.sanger.ac.uk/resources/mouse/genomes/). SNPs with a Phred score ≥ 50, identified by comparing the NCBIM37 reference sequence against the NGS NOD/ShiLtJ, were downloaded for the region spanning the *Idd18.4* and *Idd18.2* loci. SNP density plots for the BAC sequencing and NGS-determined SNPs were generated by counting the number of SNPs in 10-kb windows, sliding 2 kb at a time, and plotting the count at the midpoint of each window. For each gene in and between the *Idd18.4* and *Idd18.2* intervals, the Gene Span (maximum genomic interval [NCBIM37] required to span all transcript models) for each gene was calculated from annotations present in RefSeq (NCBIM37) (27), CCDS (Release 7) (28), UCSC (mm9, http://genome.ucsc.edu/) (29), and Ensembl and Vega (both Release 67) (30). All annotations described above were entered into the T1Dbase (31) displayed graphically using Gbrowse (32) and can be viewed at http://www.t1dbase.org.

### Identification and RNA expression of alternatively spliced *Ptpn22* transcripts

For expression studies, total RNA was extracted using TRIzol (Invitrogen) following the manufacturer’s instructions. Whole organs were immediately homogenized in TRIzol using a Polytron homogenizer, and single-cell suspensions were obtained for thymocytes before addition to TRIzol. One microgram of total RNA was then used to make cDNA using SuperScript II reverse transcriptase (Invitrogen) following the manufacturer’s instructions. To identify alternatively spliced transcripts of *Ptpn22*, M90388 was searched against the mouse expressed sequence tag (EST) database at the National Center for Biotechnology Information using the basic local alignment search tool (33). Thirteen ESTs were identified and aligned to AC124698 using est2genome (23); these predicted six novel alternatively spliced transcripts, four of which were confirmed by the ability of primers spanning the unique exon-exon boundaries to amplify spleen or kidney cDNA from NOD and line 1101 congenic mice (Supplemental Table IIB). RT-PCR on the same cDNA using a range of *Ptpn22* exonic primers that spanned few or many exons identified products with unexpected sizes that were gel extracted using the QIAquick Gel Extraction Kit (Qiagen) following the manufacturer’s instructions, and sequenced to identify nine additional alternatively spliced transcripts with additional or spliced out exons (Supplemental Table IIC). Primer and probe sets were designed to the unique regions of full-length and a selection of alternative transcripts of *Ptpn22* (Supplemental Table IID) and qPCR was used to assess the expression levels of these transcripts and β_2_-microglobulin in an ABI Prism 7300 Real-Time PCR system (Applied Biosystems) in thymocytes from 3-wk-old male mice and whole spleen from 9-wk-old female mice, both from lines 2410 and 1101. All reactions were performed using TaqMan Universal Master Mix. The expression levels are given as dCT, which is calculated by subtracting the cycle threshold value (the cycle number at which message is first detected) for β_2_-microglobulin from the cycle threshold value for each assessed transcript. Lower dCT values indicate higher RNA levels. As the cycle thresholds were detected in the exponential phase of amplification, a 1-dCT difference is equivalent to a 2-fold change, and a 4-dCT difference equals a 16-fold change in RNA levels. Differences in expression were compared using Student unpaired, two-tailed *t* test (GraphPad Prism software).

### PEP expression

Single-cell suspensions were prepared from thymi obtained from 6–8-wk-old mice. Erythrocytes were lysed using RBC lysis buffer (Sigma), and the remaining thymocytes were washed in 1x RPMI 1640 (Mediatech), pelleted, and lysed in 1x TNE (10 mM Tris [Fisher], 0.2 M NaCl [Sigma], 1 mM EDTA, pH 7.4 [Sigma]) with 0.1% NP40, 1 mM PMSF, 10 μg/ml soybean trypsin inhibitor, and 10 μg/ml aprotinin and leupeptin (all from Sigma). Equal amounts of total lysate from each mouse were loaded onto 10% Tris glycine polyacrylamide gel (Invitrogen), blotted onto nitrocellulose membrane, probed with rabbit polyclonal anti-PEP Ab (1:3000; provided by Andrew C. Chan, Genentech) or mouse anti–α-TUBULIN Ab (1:2000; Santa Cruz Biotechnology), and respective secondary Abs linked to HRP (GE Healthcare), and detected with ECL (GE Healthcare) and autoradiography films (HyBlot CL; Denville Scientific). The films were scanned, backgrounds were adjusted using Adobe Photoshop CS3, and densitometry readings were obtained using ImageJ software (34). Relative ratios of PEP to α-TUBULIN were calculated for each of the four independent blotting experiments by normalizing each ratio to the lowest ratio obtained in each experiment. The relative ratios for congenic mice with NOD- or B6-derived alleles at *Ptpn22* were pooled, and the Mann–Whitney *U* test was used to calculate statistical significance (GraphPad Prism software).

### Measuring p44-MAPK phosphorylation

Splenocytes were dissociated into single-cell suspensions and filtered through a nylon strainer (70 μm). RBCs were lysed by addition of water, followed by equal volumes of 2x PBS and RPMI 1640. Splenic T cells were isolated by negative depletion by incubation with rat anti-mouse B220 and rat anti-mouse CD11b (M1/70) Abs, followed by incubation with sheep anti-Rat IgG Dynabeads (Invitrogen). T cells were resuspended in RPMI with 5% FBS and warmed to 37°C for 5 min. For stimulation, 20 μg/ml anti-mouse CD3 (2C11) was added, followed by the addition of 20 μg/ml mouse anti-Armenian and Syrian Hamster IgG Abs (G94-56; BD Pharmingen). Cells were incubated for 2 min at 37°C and lysed immediately in 1× Cell Lysis Buffer (Cell Signaling Technology). Levels of phospho-p44 MAPK were measured by ELISA using the PathScan Phospho-p44 MAPK (Thr202/Tyr204) Sandwich ELISA Ab Pair (Cell Signaling Technology) following the manufacturer’s instructions. Samples were performed in triplicate and averaged. Statistical significance of the difference between congenic mouse strains was determined by Student unpaired, one-tailed *t* test (GraphPad Prism software).

### CD2 Ab staining and flow cytometry analysis

Single-cell suspensions were prepared from the spleen and bone marrow obtained from 8- to 12-wk-old mice. Cells were stained using B220-PerCP and CD2-FITC (both from BioLegend) and analyzed on a FACSCalibur (Becton Dickinson) using FlowJo software (Tree Star). The mean fluorescence intensity (MFI) of CD2 on various cell subsets was determined, and the Mann–Whitney *U* test was used to calculate statistical significance between groups (GraphPad Prism software).

## Results

### Identification of a B6 susceptibility allele, *Idd18.2*, located between *Idd10* and *Idd18.3*

**FIGURE 1.**
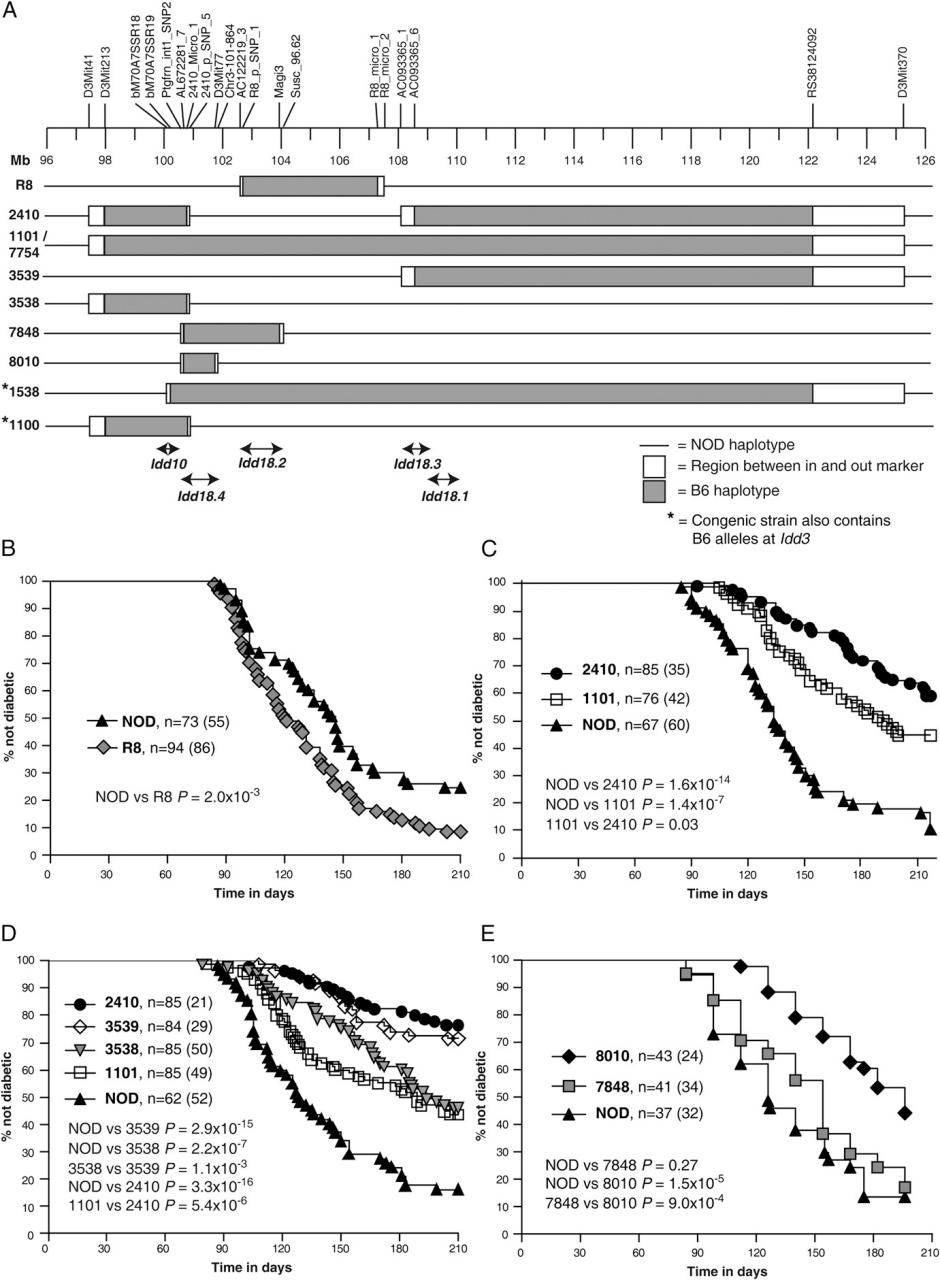
A B6 susceptibility locus, *Idd18.2*, and a B6 protective locus, *Idd18.4*, are located between *Idd10* and *Idd18*. (**A**) The congenic strains used to define *Idd18.2* and *Idd18.4* and those used in the T1D frequency studies are shown. Lines R8 and 2410 confirm the existence of the B6 susceptibility locus, which was refined to between the centromeric recombination point of R8 and the telomeric recombination point of line 7848, and is a 1.31-Mb locus between, but not including, the microsatellite markers *AC122219_3* and *Susc_96.62*. *Idd18.4* is located between the centromeric and telomeric recombination points of line 8010, and it is a 953-kb locus between, but not including, the microsatellite markers *Ptgfrn_Int1_SNP2* and *Chr3-101,864*. The locations of markers in NCBIM37 are shown. (**B**) The diabetes frequency study (conducted for 210 d in 1995–1996) indicating that congenic mice from line R8, which have a single B6 introgressed segment between *Idd10* and *Idd18*, are more susceptible to T1D compared with NOD mice (*p =* 2.0 × 10^−3^). This finding suggests the presence of a B6 susceptibility locus between *Idd10* and *Idd18.3*. (**C**) The diabetes frequency study (conducted for 214 d in 2003–2004) indicating that line 2410 congenic mice, which have B6-derived alleles at *Idd10* and *Idd18* but NOD-derived alleles at *Idd18.2*, are more protected from T1D compared with line 1101 (*p =* 3.0 × 10^−2^). Line 1101 differs from line 2410 by the presence of B6-derived alleles at *Idd18.2*. Therefore, line 2410 confirms the presence of a B6 susceptibility locus between *Idd10* and *Idd18.3*. (**D**) The diabetes frequency study (conducted for 210 d in 2006–2007) of lines 3538 and 3539 assessing the protection associated with the *Idd10* and *Idd18* loci alone without B6 susceptibility alleles at *Idd18.2*. Lines 3538 and 3539 were much more protected from diabetes compared with NOD (*p =* 2.2 × 10^−7^, 2.9 × 10^−15^, respectively), indicating that the low levels of T1D protection observed in congenic strains with either *Idd10* or *Idd18* in combination with B6-derived alleles at *Idd18.2* in previous congenic strain mapping studies were due to the B6 *Idd18.2* susceptibility alleles masking the protection associated with *Idd10* or *Idd18*, respectively. The diabetes frequencies of lines 2410 and 1101 were also repeated in this experiment. The protection associated with the NOD allele of *Idd18.2* is clearly observed again in line 2410, as it is much more protected from diabetes than line 1101 (*p =* 5.4 × 10^−6^). The data for 3538 and NOD shown in this panel have been published previously (22). (**E**) The diabetes frequency study (conducted for 196 d in 2010–2011) of lines 7848 and 8010 confirm the candidacy of *Ptpn22* and identify an additional *Idd* locus, *Idd18.4*, containing the candidate *Cd2*. Line 7848 and 8010 share a small introgressed B6 DNA segment in *Idd18.4*, but have B6- or NOD-derived alleles at *Ptpn22*, respectively. Line 7848 has a higher frequency of diabetes compared with line 8010, confirming the candidacy of *Ptpn22.* Moreover, the high level of diabetes protection associated with line 8010 identifies the additional B6 protective locus, *Idd18.4*. *n* = the number of mice in each cohort; numbers in parentheses indicate mice that developed diabetes by the end of the study.

The previously published congenic strain mapping of the *Idd10* and *Idd18* loci detailed in Podolin et al. (5) involved the development of several congenic strains including one not reported in that study, line R8. This strain was homozygous for a single B6 introgressed segment located between *Idd10* and *Idd18.3* (Fig. 1A) and, unexpectedly, was more susceptible to T1D than NOD mice (*p* = 2.0 × 10^−3^; Fig. 1B). We, therefore, hypothesized that a B6 susceptibility allele, designated *Idd18.2*, exists between *Idd10* and *Idd18.3* in the introgressed segment present in line R8. To confirm the existence of *Idd18.2*, we developed a new congenic mouse strain, line 2410, which has NOD alleles at *Idd18.2* and is homozygous for two B6 introgressed segments: one spanning *Idd10* and the second spanning *Idd18.3* and *Idd18.1* (Fig. 1A). Line 2410 mice have a lower T1D frequency (*p* = 3.0 × 10^−2^; Fig. 1C) compared with the control strain, line 1101, which is homozygous for a single B6 introgressed segment spanning *Idd10*, *Idd18.2*, *Idd18.3*, and *Idd18.1* (Fig. 1A). The T1D frequency comparison of lines 1101 and 2410 was repeated in a later study and produced the same results (Fig. 1D; 1101 versus 2410, *p* = 5.4 × 10^−6^). As lines 1101 and 2410 differ only at *Idd18.2*, these results confirm that *Idd18.2* is a B6 susceptibility locus, which can reduce the protection associated with the combined B6-derived alleles of *Idd10*, *Idd18.3*, and *Idd18.1*.

### The *Idd18.2* B6 susceptibility alleles partially mask the protection associated with the B6-derived alleles of *Idd10*, or *Idd10* and *Idd18.1*

The congenic strain mapping of *Idd10* and *Idd18* (now known to be composed of two loci, *Idd18.3* and *Idd18.1*) (7) has always been performed by truncating the *Idd10* or *Idd18* loci present in line 1101, NOD.B6 *Idd10 Idd18.2 Idd18*, or line 1538, NOD.B6 *Idd3 Idd10 Idd18.2 Idd18*. Truncation of either the centromeric *Idd10* locus or the telomeric *Idd18* locus reduced the T1D frequency to a NOD-like or nearly NOD-like frequency for those derived from line 1101 (5), and *Idd3*-like frequency for those derived from line 1538 (4). Our interpretation at the time was that the truncated congenics contained only a single *Idd* locus, either *Idd10* or *Idd18*, in those derived from line 1101, or two *Idd* loci, either *Idd3* and *Idd10* or *Idd3* and *Idd18*, in those derived from line 1538, and that both *Idd10* and *Idd18* were required in combination to observe substantial T1D protection, and either locus alone did not confer protection. However, with the existence of the B6 susceptibility allele *Idd18.2*, located between *Idd10* and *Idd18* now evident in the current study, we now know that what we thought were isolated *Idd10* and *Idd18* congenic segments, with or without *Idd3*, actually also contained the B6 susceptibility alleles of the *Idd18.2* locus. To observe the protection of isolated *Idd10* and *Idd18* loci without the presence of the B6 susceptibility alleles at *Idd18.2*, we separated the *Idd10* and *Idd18* DNA segments present in line 2410 and performed a frequency study on the resulting NOD.B6 *Idd10* (line 3538) and NOD.B6 *Idd18* (line 3539) congenic strains (Fig. 1A). Both congenic strains, NOD.B6 *Idd10* and NOD.B6 *Idd18*, were highly protected from T1D as compared with NOD (*p* = 2.2 × 10^−7^, 2.9 × 10^−15^, respectively; Fig. 1D). Remarkably, the *Idd18* locus present in line 3539 provided as much T1D protection as the combination of the isolated *Idd18* and *Idd10* segments (line 2410). This indicates that in the previous studies, where we had observed negligible T1D protection in the congenic strains with B6-derived alleles at *Idd10* and *Idd18.2* or *Idd18.2* and *Idd18* that the B6-derived *Idd18.2* susceptibility alleles had been masking the protective effects of the *Idd10* and *Idd18* protective loci.

### Novel congenic strains, lines 7848 and 8010, help refine *Idd18.2* to a 1.31 Mb locus and confirm the candidacy of *Ptpn22*

Of the two congenic strains (lines R8 and 2410) that delineate the *Idd18.2* locus, line R8 has the smaller locus and, thus, defines *Idd18.2* as a 4.8-Mb locus between, but not including, the microsatellite markers *AC122219_3* and *R8_micro_2* (Fig. 1A). This locus contains 71 genes, the most centromeric and telomeric being *Nr1h5* and *Ahcy11*, respectively. At least seven of these are involved in autoimmune diseases or immune cell function: *Ptpn22*, *Adora3*, *Rbm15*, *Lrig2*, *Cd53*, *Nras*, and a cluster of six chitinase-like genes. *Ptpn22* is the most striking candidate, as the human ortholog, *PTPN22*, is associated with several autoimmune diseases (35).

To confirm the positional candidacy of *Ptpn22*, we developed two new congenic strains, lines 7848 and 8010, which are homozygous for B6 introgressed segments in *Idd18.2* that share the same centromeric recombination point, but that differ at the telomeric recombination point, resulting in lines 7848 and 8010 having B6- and NOD-derived alleles at *Ptpn22*, respectively (Fig. 1A). Line 7848 congenic mice, which have B6 alleles at *Ptpn22*, have a higher frequency of T1D than line 8010 does, which has NOD alleles at *Ptpn22* (*p =* 9.0 × 10^−4^; Fig. 1E). These results indicate that line 7848 contains the *Idd18.2* B6 susceptibility locus and, therefore, confirms the candidacy of *Ptpn22*.

**FIGURE 2.**
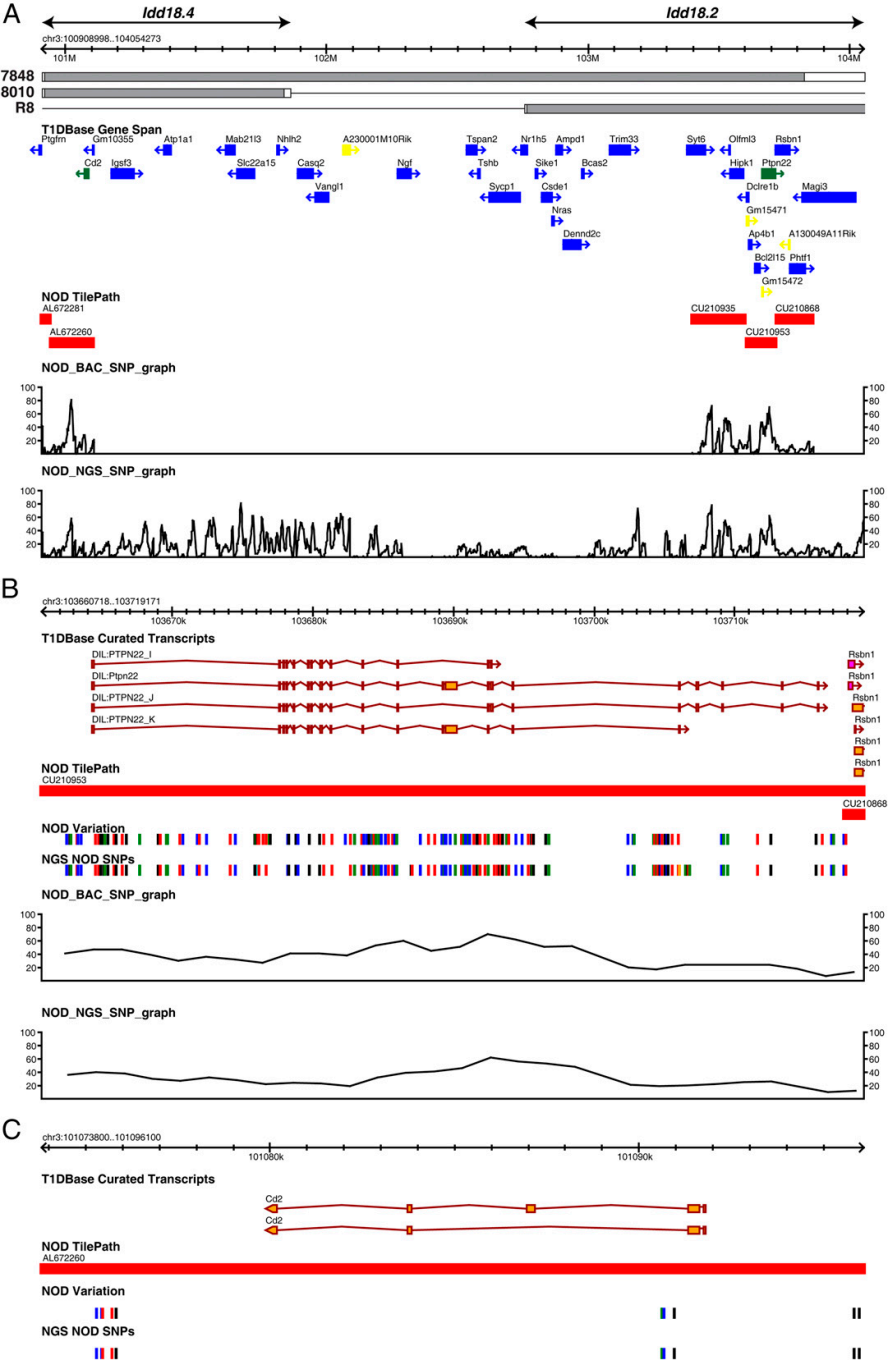
*Idd18.2* and *Idd18.4* annotation and sequence polymorphisms in NCBIM37. (**A**) The congenic boundaries of line 8010 define the *Idd18.4* locus, and the centromeric boundary of line R8 and the telomeric boundary of line 7848 define the *Idd18.2* locus. The T1DBase Gene Span track displays the maximum genomic interval for each gene: candidate genes are displayed in green, blue indicates other protein-encoding genes, and yellow indicates small cytoplasmic RNA genes. The NOD TilePath track represents the sequenced NOD BAC clones, and the NOD_BAC_SNP_graph represents the SNP density per 10 kb, detected by comparing the NOD BAC clone sequence to the B6 reference sequence. The NOD_NGS_SNP_graph displays the SNP density per 10 kb of SNPs detected between the NCBIM37 B6 reference sequence and NOD/ShiLtJ NGS data. (**B** and **C**) The coding sequences of the transcripts from the *Idd18.2* and *Idd18.4* candidate genes *Ptpn22* and *Cd2* are shown, respectively, in the T1DBase Curated Transcripts track. The SNPs surrounding these genes are shown underneath. These SNPs have been detected by comparing the B6 reference sequence to either the NOD BAC clone sequence (NOD Variation track) or the NOD/ShiLtJ NGS data (NGS NOD SNPs track). Black, red, blue, and green lines represent G, T, C, and A NOD alleles, respectively. The NGS data also contain ambiguous bases. R, Y, M, K, W, and S are represented as brown, orange, pink, cyan, gold and gray lines, respectively. Yellow lines are SNPs where the base information is unknown. Note that where multiple SNPs are located close together in these two tracks, the lines in the NOD variation or NGS NOD SNPs track may represent more than one SNP. There is a higher SNP density over *Ptpn22*; to represent this density better, the NOD_BAC_SNP_graph and NOD NGS_SNP_graph display the density of SNPs per 10 kb.

The locus delineated by lines 7848 and 8010 (Fig. 1A) overlaps with the *Idd18.2* locus delineated by line R8 (Fig. 1A). As both loci contain *Idd18.2*, the *Idd18.2* locus is, therefore, located between the centromeric recombination point in line R8 (between *AC122219_3* and *R8_p_SNP_1*) and the telomeric recombination point in line 7848 (between *Magi3* and *Susc_96.62*; Fig. 1A). Therefore, *Idd18.2* is a 1.31-Mb locus between, but not including, the microsatellite markers *AC122219_3* and *Susc_96.62* (Fig. 1A), and contains only 18 protein-encoding genes, including the candidate *Ptpn22* (Fig. 2A).

### Novel congenic strains, lines 7848 and 8010, also identify a second Idd locus, *Idd18.4*

The diabetes protection provided by the small B6 congenic segment in line 8010 (Fig. 1A) as compared with NOD control mice was unexpected (Fig. 1E), and it leads to the localization of an additional B6 protective *Idd* locus, designated *Idd18.4*, located between *Idd10* and *Idd18.2* (Fig. 1A). *Idd18.4* is defined by the centromeric (*Ptgfrn_Int1_SNP2* and *AL672281_7*) and telomeric (*D3Mit77* and *Ch3:101,864*) recombination points in line 8010 to a 953-kb locus between, but not including, markers *Ptgfrn_Int1_SNP2* and *Ch3:101,864*. The centromeric recombination point of *Idd18.4* and the telomeric recombination point of *Idd10* (22) are located between the same polymorphisms, *Ptgfrn_Int1_SNP2* and *AL672281_7.* The B6 and NOD BAC clone sequence between these polymorphisms is identical in NOD and B6. Therefore, the NOD/B6 polymorphisms present in the *Idd10* and *Idd18.4* loci are distinct, and the protection observed for line 8010 is due to *Idd18.4* alone and not *Idd10*. *Idd18.4* contains eight genes (Fig. 2A), including the candidate *Cd2*. The interactions between CD2 and the murine and the human ligands (CD48 and CD58, respectively) are important for immune function. These genes are associated with several human autoimmune diseases, including multiple sclerosis and rheumatoid arthritis, and with a murine model of lupus (18–20).

### Resequencing of *Ptpn22* reveals a high degree of variation between NOD and B6, but excludes an analogous R620W human polymorphism

In humans, the *PTPN22* variant associated with susceptibility to autoimmune disease is a nonsynonymous SNP, R620W, in exon 14, which encodes a proline-rich region that interacts with C-terminal Src kinase. We therefore tested the possibility that an analogous polymorphism was present in *Ptpn22.* To identify any sequence polymorphisms in *Ptpn22* between NOD and B6, three NOD BAC clones spanning *Ptpn22* (Fig. 2A) were selected and sequenced at the WTSI. Mouse *Ptpn22* spans 52 kb and consists of 21 exons. All the donor and acceptor splice sites of mouse and human *PTPN22* genes are in agreement with the canonical GT-AG splice site except for the splice site between mouse exons 13 and 14 that contains a noncanonical GC-AG splice site (36). Six SNPs between NOD and B6 were identified in the coding sequence of *Ptpn22* (Supplemental Table IIA), five of which were synonymous and one nonsynonymous SNP in exon 12, none of which were analogous with R620W. The nonsynonymous SNP (rs33557973) V319I is located in the interdomain region of PEP between the phosphatase domain and PEST-rich region, which is involved in interactions with various ligands (37). A multiple-species alignment of LYP/PEP indicates that this residue is present in an unconserved region and is conserved as a hydrophobic aliphatic residue in most species (Supplemental Fig. 1). As valine and isoleucine are both hydrophobic aliphatic residues and are similar, we believe it is unlikely that V319I will alter the structure or phosphatase activity of PEP. An additional 177 SNPs were identified in the noncoding regions of *Ptpn22* (Fig. 2B). Although polymorphisms were not identified in the core promoter region or 3′ untranslated region (UTR) of *Ptpn22*, many SNPs are located in polypyrimidine tracts and branch site sequences and, therefore, could affect splicing (38, 39); they are also located in putative downstream sequence elements that could affect polyadenylation (40).

### *Ptpn22* alternatively spliced transcripts are differentially expressed between NOD- and B6-derived alleles

**FIGURE 3.**
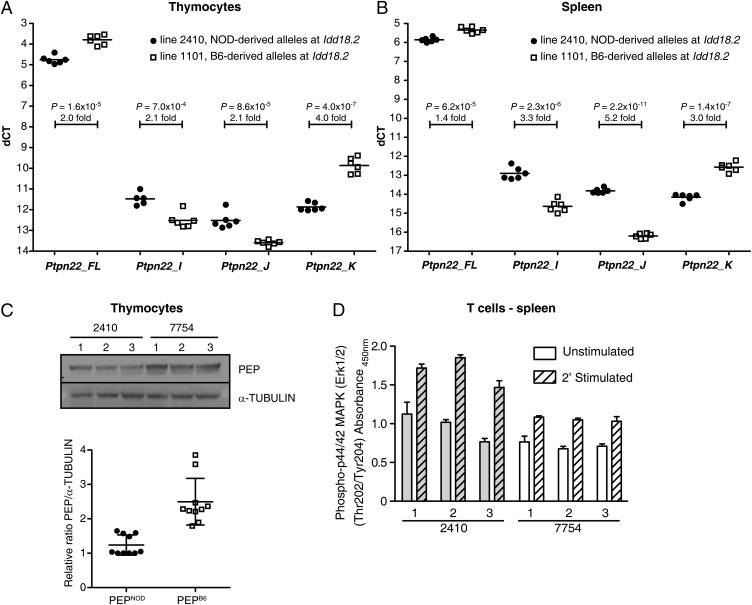
Differential expression by genotype of full-length *Ptpn22* mRNA and PEP protein. (**A**) Gene expression levels are displayed as dCT (see *Materials and Methods*); lower dCT values represent higher expression. Thymocytes isolated from male 3-wk-old line 1101 congenic mice (B6-derived alleles at *Ptpn22*) express 2-fold more full-length *Ptpn22* mRNA compared with line 2410 congenic mice (NOD-derived alleles at *Ptpn22*). A similar genotype dependent expression is observed for the alternative spliced transcript, *Ptpn22_K*. The alternatively spliced transcripts *Ptpn22_I* and *Ptpn22_J* have the opposite genotype dependent expression, with line 2410 having the greater expression. *n* = 6. (**B**) A similar, albeit slightly lower, pattern of expression is observed for the transcripts in whole spleen from line 1101 and 2410 congenic mice: full-length *Ptpn22* mRNA from line 1101 is expressed 1.4-fold more compared with line 2410. *n* = 6. (A and B) Results are representative of at least two independent experiments. (**C**) The protein expression of PEP follows the pattern of the mRNA expression. A representative Western blot analysis of P56 mouse thymocyte total lysates from line 7754 (new designation for 1101) and line 2410 congenic mice, probed against PEP and α-TUBULIN is shown. Each lane represents thymocytes collected from an individual animal. Relative ratios of PEP to α-TUBULIN were collected from individual P45-P56 mouse thymocyte total lysates in four independent experiments performed on different days by normalizing each ratio to the lowest ratio collected in each experiment. In the scatter plots, the horizontal bars represent the mean and lines represent the SD. Mann-Whitney was used to calculate statistical significance. PEP is expressed 2-fold higher in congenic mice with B6-derived alleles at *Ptpn22* (lines 7754 and 7848) compared with congenic mice with NOD-derived alleles at *Ptpn22* (lines 2410 and 8010; *p* = 2.0 × 10^−4^). *n* = 10. (**D**) Higher PEP expression is associated with increased negative regulation of TCR signaling. Line 7754 congenic mice with B6-derived alleles at *Idd18.2* have ∼1.6-fold lower levels (*p* = 0.026) of phosphorylated MAPK compared with line 2410 congenic mice with NOD-derived alleles at *Idd18.2* following stimulation. Each set of bars represents splenic T cells from an individual animal that were either unstimulated (unhatched bars) or stimulated with anti-CD3 Ab plus a cross-linker for 2 min (hatched bars). Phospho-p44 MAPK levels were assessed in cell lysates from 2410 (gray bars) and 7754 (open bars) congenic mice by ELISA. Three animals were tested for each congenic strain. Error bars are the SD of triplicates. The levels of phospho-p44 MAPK after stimulation were compared using Student one-tailed, unpaired *t* test.

To determine whether the polymorphisms present between NOD and B6 could affect the splicing and expression of *Ptpn22*, we first searched for alternatively spliced *Ptpn22* transcripts in EST databases and by RT-PCR. The expression of 13 different alternatively spliced transcripts (Supplemental Table IIB, IIC) was confirmed (data not shown); a contributing factor to this large number may be the rare GC-AG splice site, as noncanonical splice sites are associated with higher levels of alternatively spliced transcripts (41). The expression of the *Ptpn22* transcripts from NOD- and B6-derived alleles at *Idd18.2* was compared using qPCR. In addition to full-length *Ptpn22*, three transcripts were found to be differentially expressed: *Ptpn22_I*, *Ptpn22_J*, and *Ptpn22_K* (Fig. 3A). *Ptpn22_I* contains all exons apart from exons 12 and 13, resulting in a change of reading frame in exon 14 and a premature termination codon in exon 15. *Ptpn22_J*, like *Ptpn22_I*, excludes exons 12 and 13 but contains a novel exon 14 that includes the first 13 nucleotides in intron 14; this inclusion returns the reading frame to that of full-length *Ptpn22* in exon 15. *Ptpn22*_*K* contains all exons apart from exon 15; this results in a change of reading frame and a premature termination codon in exon 17 (Fig. 2B). Full-length *Ptpn22* is expressed 2-fold and 1.4-fold higher in line 1101 compared with line 2410 in thymocytes from 3-wk-old male mice (*p* = 1.6 × 10^−5^) and whole spleen from 9-wk-old female mice (*p* = 6.2 × 10^−5^), respectively (Fig. 3A, 3B), indicating that B6-derived alleles of *Idd18.2* are more highly expressed than NOD-derived alleles. In the same tissue samples, the *Ptpn22_I*, _*J* and _*K* alternatively spliced transcripts are expressed ∼50–100-fold less than full-length *Ptpn22*. *Ptpn22*_*K* has a similar genotype-dependent expression, with the B6-derived alleles in line 1101 having a higher expression (3–4-fold) compared with line 2410. Conversely, *Ptpn22*_*I* and *Ptpn22*_*J* have the opposite genotype-dependent expression with B6-derived alleles expressed 2.1–5.2-fold lower in line 1101 compared with NOD-derived alleles in line 2410 (Fig. 3A, 3B).

### Susceptible B6-derived *Ptpn22* alleles have higher protein expression levels and decreased TCR signaling

To determine whether the differential expression of *Ptpn22* mRNA resulted in differential protein levels, we measured the amount of PEP in thymocyte lysates from line 7754 (new designation for line 1101) and 7848 congenic mice, which have B6-derived alleles at *Ptpn22*, and compared this to line 2410 and 8010 congenic mice that have NOD-derived alleles at *Ptpn22*. We found the same differential expression pattern of PEP as observed for full-length *Ptpn22* transcripts: B6-derived *Idd18.2* alleles result in 2-fold higher PEP expression as compared with the NOD-derived alleles (*p* = 2.0 × 10^−4^; Fig. 3C). Since LYP/PEP is known to regulate TCR signaling negatively (42, 43), we examined whether the difference in PEP expression would affect TCR signaling by examining the levels of induced phosphorylation of MAPK in splenic T cells following activation. We found that B6-derived *Idd18.2* alleles present in line 7754 congenic mice had ∼1.6-fold lower levels (*p* = 0.026) of p44 MAPK phosphorylation after stimulation compared with the NOD-derived alleles in 2410 congenic mice (Fig. 3D)

### NOD-derived susceptibility alleles at *Idd18.4* result in lower CD2 expression on B cells

As *Cd2* is the candidate gene for *Idd18.4*, we investigated whether potentially functional polymorphisms were present between the NOD and B6 alleles. The genes encoding CD2 in human and mouse are similar; both span ∼12 kb and consist of five exons, and all the donor and acceptor splice sites are in agreement with the canonical GT-AG splice site. Genomic sequence from B6 and NOD BAC clones spanning *Cd2* was publicly available because of the mapping and sequencing of the *Idd10* locus (22, 44) and these were aligned to identify polymorphisms (Fig. 2C). Although polymorphisms were not detected in the coding sequence, 5′ or 3′ UTRs nor splice acceptor and donor sites, excluding an obvious structural difference between NOD and B6, four SNPs and six insertion–deletion polymorphisms were identified in the introns of *Cd2*, and four microsatellite polymorphisms were detected within 2.5 kb upstream of the 5′ UTR (Supplemental Table III), all of which could affect the expression or splicing of *Cd2*.

**FIGURE 4.**
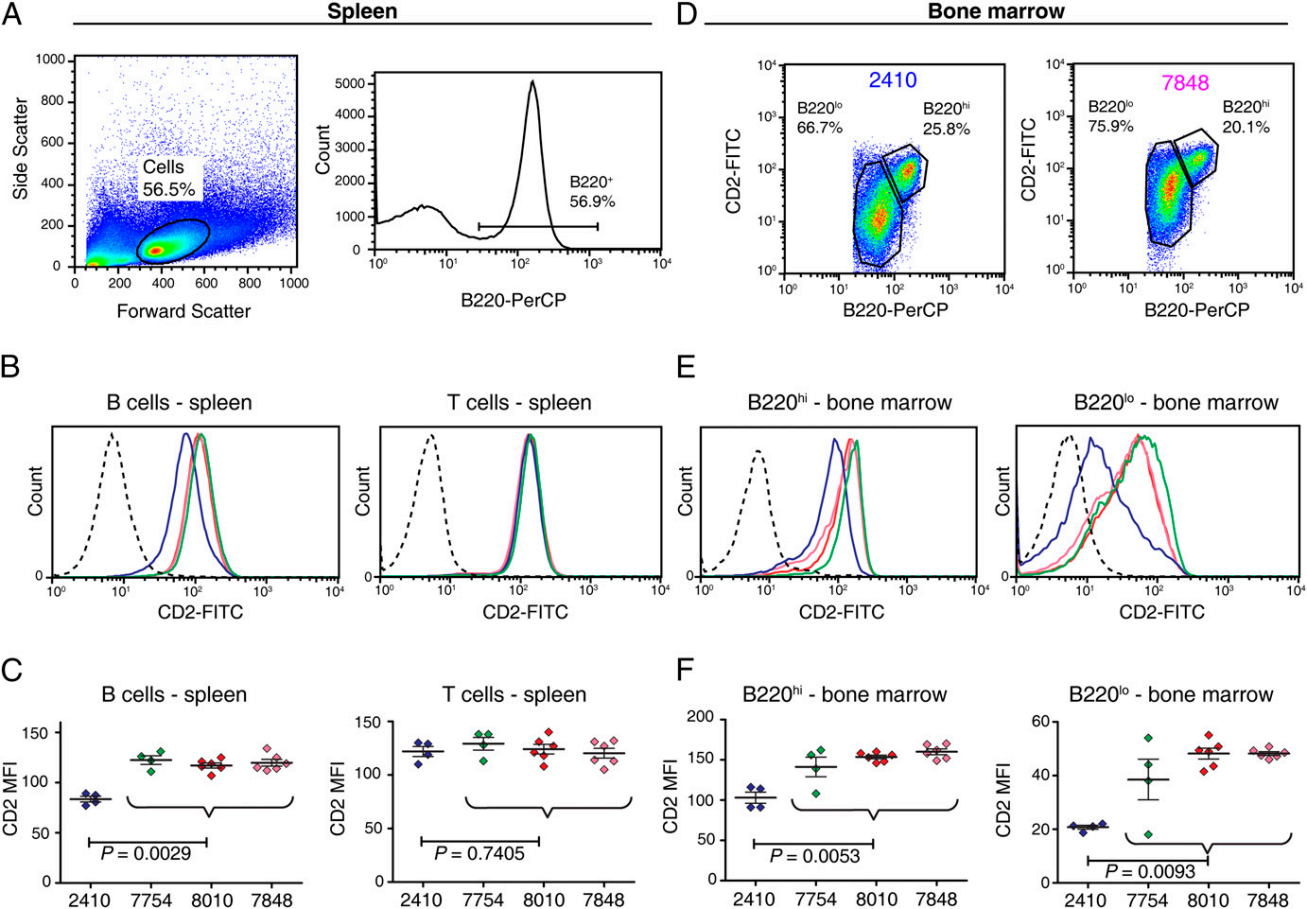
*Cd2* genotype determines CD2 expression levels on B cells in spleen and bone marrow. (**A**) Light scatter of the splenic cells and histogram showing the gating on the splenic B220^+^ cells. (**B**) Histograms showing the levels of CD2 expression on the splenic B cells (B220^+^) and T cells (non B220^+^). In (B) and (**E**), staining with an isotype control mAb is represented by the black dotted line. In (B), (**C**), (E), and (**F**), lines 2410, 7754, 8010, and 7848 are represented by blue, green, red, and pink, respectively. (C) Scatter plots showing the MFI of CD2 on splenic B and T cells for the four strains. Thick and thin, horizontal, black lines represent the mean and the SEM, respectively. MFIs were compared based on genotype at *Idd18.4*, and the Mann–Whitney *U* test was used to determine whether these were statistically significant. (**D**) B220 and CD2 staining on bone marrow lymphocytes from 2410 and 7848 show the two populations, B220^lo^ and B220^hi^, analyzed. (E) Histograms of CD2 expression on the B220^hi^ and B220^lo^ bone marrow populations comparing the four strains. (F) Scatter plots showing the MFI of CD2 on bone marrow-derived B220^lo^ and B220^hi^ populations for the four strains. Thick and thin, horizontal, black lines represent the mean and the SEM, respectively. MFIs were compared based on genotype at *Idd18.4*, and the Mann–Whitney *U* test was used to determine whether these were statistically significant. In addition to the data shown in this figure, we also performed two additional, separate experiments in which we tested mice with B6- or NOD-derived alleles at *Idd18.4* in splenic B cells (*n* = 19 for each genotype) and in B220^hi^ and B220^lo^ bone marrow cells (*n* = 11 for each genotype). We observed the same expression differences as shown in this figure.

To determine whether CD2 was expressed differently in congenic mice with B6- or NOD-derived alleles at *Idd18.4*, we investigated the cell surface expression of CD2 on immune cells from the spleen and bone marrow in line 2410 congenic mice, which have NOD-derived alleles at *Idd18.4*, and in line 7754, 7848, and 8010 congenic mice that have B6-derived alleles at *Idd18.4*. We found that splenic B cells from congenic mice with B6-derived *Idd18.4* alleles had a 1.4-fold higher CD2 surface expression compared with mice with NOD-derived alleles at *Idd18.4* (*p* = 2.9 × 10^−3^; Fig. 4A–C). A similar expression difference was observed in B220^hi^ (mature) and B220^lo^ (immature) bone marrow derived cells, with congenic strains with B6-derived alleles at *Idd18.4* having 1.6-fold (*p* = 5.3 × 10^−3^) and 1.9-fold (*p* = 9.3 × 10^−3^) higher cell surface expression of CD2, respectively, compared with mice with NOD-derived *Idd18.4* alleles (Fig. 4D–F). However, this expression difference was not observed for splenic T cells (Fig. 4B, 4C), and was also not observed when we looked specifically at CD4^+^ and CD8^+^ splenic T cell subsets (data not shown), suggesting that this expression difference is specific to B cells.

## Discussion

### Identification of two novel Idd loci, *Idd18.2* and *Idd18.4*, on chromosome 3

Using congenic strain mapping, we have identified two novel *Idd* loci, *Idd18.2* and *Idd18.4*, both of which are located between *Idd10* and *Idd18.3* on chromosome 3. *Idd18.4* is located immediately telomeric to *Idd10* and is a 953-kb locus, containing eight genes, in which the B6-derived alleles confer T1D protection. Conversely, the B6-derived alleles of the 1.31 Mb *Idd18.2* locus, which contains 18 protein-encoding genes, confer T1D susceptibility. Although many of the genes in these two loci have polymorphisms between B6 and NOD, and are therefore positional candidates for altering susceptibility to T1D, we focused on *Ptpn22* and *Cd2* because these genes or the signaling pathways they are involved in have been associated with several human autoimmune diseases (18, 19, 35). Although we did not identify polymorphisms in either gene that would be expected to affect protein function, we did find several polymorphisms likely to affect gene expression, and we went on to identify ∼2-fold higher RNA and protein expression of *Ptpn22*/PEP in mice with susceptible B6-derived alleles at *Idd18.2* and ∼1.5-fold higher CD2 expression in mice with protective B6-derived alleles at *Idd18.4*. Furthermore, we observed that mice with susceptible alleles at *Idd18.2* have decreased signaling downstream of the TCR upon immune stimulation, a phenotype consistent with an increased level of a negative regulator of signaling, such as the PEP phosphatase encoded by *Ptpn22*. Differential expression by the B6 and NOD *Ptpn22* alleles of alternatively spliced mRNAs was also observed, but the presence of protein from these transcripts and their potential influences on disease susceptibility were not investigated. It is important to note that despite the expression differences observed for *Ptpn22* and *Cd2*, they remain only candidate genes for their regions. Other polymorphic genes within the regions could be responsible for the change in autoimmune disease susceptibility.

### Challenges of fine mapping *Idd* loci with congenic strain mapping

As exemplified in this study, the use of congenic strain mapping in locating *Idd* and other disease associated loci in the mouse has been highly successful, and in many cases the original loci identified appear to be constituted of several smaller clustered loci. Two main challenges arise in the fine mapping of these clustered loci—namely, the development of congenic strains with much smaller introgressed segments containing, ideally, only the putative candidate gene to confirm the candidacy by exclusion of all others, and the complex genetic interactions that can occur between loci and can mask or reveal protection associated with a particular allele.

Congenic strain mapping has proved less successful in the fine-mapping of loci because the production of congenic mice is dependent on recombination, which does not occur evenly throughout the genome but has observable regions of hot spots, typically 1–2 kb long, surrounded by regions lacking recombination that can extend 10–100 kb or more (45). For instance, after identifying the candidate gene *Ptpn22* using line 2410, we wanted to develop a congenic strain with as small an introgressed B6 region as possible containing *Ptpn22*, but excluding all neighboring candidate genes, or at least those known to function in immune cells. We were able to find a mouse with a suitable recombination point telomeric to *Ptpn22*. However, after genotyping nearly 1000 mice, we did not find our desired additional recombination event centromeric to *Ptpn22* that would exclude *Cd2*. Therefore, we continued with the most informative recombination events detected, which resulted in lines 7848 and 8010, the refinement of *Idd18.2*, and serendipitously identified *Idd18.4*. A similar inability to obtain a desired recombination event was observed during the fine-mapping of the *Idd3* locus, where it was not possible to find a recombination event between the *Il2* and *Il21* genes (46). Congenic strains with single introgressed genes at *Ptpn22* or *Cd2* would be useful tools to confirm the candidacy of these functional candidates; however, it seems improbable that these could be developed. Different technologies, such as gene targeting, will need to be used to confirm the candidate genes. However, if the gene-targeting constructs are generated in cells not derived from NOD mice, the experimental approach is still limited by recombination events because of carryover of neighboring non-NOD genetic material.

Complex genetic interactions have been observed to occur between clustered loci. In this article, we report that B6-susceptible alleles at *Idd18.2* masked the protection associated with B6 protective alleles at *Idd10* or *Idd18.3/18.1*. This masking became apparent when comparing the low levels of T1D protection observed in NOD.B6 *Idd10*, *18.2* (T1D frequency similar to NOD) and NOD.B6 *Idd18.2*, *18.3*, *18.1* (T1D frequency slightly higher than NOD) congenic strains published in a previous study (5) (see strains R3 and R6, respectively) with the high levels of T1D protection observed in this study for line 3538, NOD.B6 *Idd10*, (T1D frequency similar to line 1101) and line 3539, NOD.B6 *Idd18.3*, *18.1* (T1D frequency similar to line 2410; Fig. 1D), where the levels of T1D protection of *Idd10* and *Idd18.3/18.1* were assessed without the presence of B6 susceptibility alleles at *Idd18.2*. Interestingly, in the previous study, the interaction between *Idd10* and *Idd18.3/18.1* was believed to be additive because both the *Idd10* and *Idd18.3/18.1* alleles were required in combination to observe any protection from T1D as either locus alone provided negligible protection (5). However, because of the identification of the *Idd18.2* locus in the current study, we now know that this is incorrect and that the genetic interaction between *Idd10* and *Idd18.3/18.1* as observed when comparing lines 2410, 3538, and 3539 appears to be nonadditive, because the level of T1D protection derived from *Idd10/18.3/18.1* (line 2410) is the same as that of *Idd18.3/18.1* (line 3539) and not greater, as would be expected from an additive interaction. Moreover, this result highlights the importance of the congenic context in which *Idd* loci are mapped because of the effects of masking. A comparison of *Idd10/18.3/18.1* (line 2410) with *Idd18.3/18.1* (line 3539) alone would suggest that the *Idd10* locus does not confer protection against T1D. However, we can see from comparing NOD and NOD.B6 *Idd10* (line 3538) that it does confer protection from T1D. In this genetic context, *Idd18.3/18.1* masks the protection associated with *Idd10*. We have previously observed the importance of congenic context in the mapping of other *Idd* loci: the protection associated with the B6-derived alleles of the *Idd18.1* locus is observed only when B6-derived alleles are present at *Idd3* (7), suggesting that NOD-derived alleles at *Idd3* mask the protection associated with B6-derived alleles at *Idd18.1*. Interestingly, as line 3539 (*Idd18.3/18.1*) has NOD-derived alleles at *Idd3*, this could imply that the protection associated with line 3539 may be due solely to the *Idd18.3* locus, or it could be that NOD-derived alleles at *Idd3* mask the protection associated with *Idd18.1* only in the context of other *Idd* loci that are not present in line 3539. Individual *Idd18.3* and *Idd18.1* congenic strains would need to be developed to address this issue. Similar complex genetic interactions have also been reported for the cluster of *Idd* loci on chromosome 1; in this case, the susceptible alleles of *Idd5.4* are apparent only in the context of susceptibility alleles at *Idd5.1/Ctla4*. Most of the genetic interactions between *Idd* loci have been identified by chance through the various congenic mapping techniques used to fine-map loci and are, therefore, more likely to be detected for clustered loci. However, genetic interactions can occur between nonclustered loci (47). A detailed knowledge of how *Idd* loci interact would be beneficial in understanding the complete interplay between different genes and susceptibility to T1D. However, it would be prohibitively expensive and time-consuming to perform combinatorial analyses using congenic strains to determine how all the known (>30) *Idd* loci interact with each other.

### *Ptpn22* is a strong candidate for *Idd18.2*

*Ptpn22* is a strong candidate for causing the susceptibility associated with the B6-derived alleles of *Idd18.2*, because the human homolog *PTPN22* contains a nonsynonymous R620W polymorphism that is associated with several autoimmune diseases, including T1D. The protein product of *PTPN22*/*Ptpn22*, LYP/PEP, downregulates immune signaling by dephosphorylating positive regulatory tyrosine residues on several of the key molecules involved in initiation of immune signal transduction, including ZAP-70, TCR-ζ chain, LCK, and FYNT (42, 48). In addition, LYP/PEP associates with the potent negative regulator of T cell signaling, C-terminal Src kinase, which also helps to downregulate TCR signaling by phosphorylating the negative regulatory Tyr505 residue on LCK (35); evidence in mice, but not humans, suggests that this interaction is synergistic (42, 49, 50). Recent studies suggest that PEP functions to regulate TCR signaling based on the strength of binding between MHC-cognate ligand and TCR. PEP can downregulate TCR signaling in the context of weak or self-antigens in naive, effector and memory T cells (51). This negative regulatory function of LYP/PEP is clearly observed in the phenotype of *Ptpn22* knockout mice in which activated T cells have a growth advantage, restimulated effector T cells have increased proliferation and cell signaling, and older mice have increased numbers of CD4^+^ and CD8^+^ effector and memory T cells (43). The effect of reduced or eliminated *Ptpn22* expression in mice on autoimmune susceptibility varies according to the target tissue and the effector mechanisms used and may depend on the genetic background of the mouse strain. Several studies have demonstrated increased percentages of FOXP3^+^ CD4^+^ T cells in the periphery and reduced organ-specific autoimmune disease susceptibility, including T1D in the NOD model (52–54). These results are consistent with our current observations of the NOD *Ptpn22* allele associated with *decreased* PEP expression and *decreased* type 1 diabetes in the NOD.B6 *Idd10*, *Idd18.3*, *Idd18.1* congenic strain. However, given the high level of sequence polymorphism and evolutionary distance between the B6 and NOD *Ptpn22* alleles (Fig. 2B), it is unlikely that the only functional change between them is a change of baseline expression levels in T cells. Regulation of the expression of *Ptpn22* in immune cell types other than those examined in this study, especially following activation, could be responsible for the disease association. We also note the further complexity that a disease allele associated with susceptibility to one or more autoimmune disease can actually be protective for a different autoimmune disease, which is the case for the human R620W polymorphism in human LYP, where the rare 620W variant is associated with protection from Crohn disease, but with susceptibility for most autoimmune diseases where association has been discovered (11). Similarly, autoimmunity in mouse models has been increased by knocking out *Ptpn22* when disease is driven via Abs (50, 55).

The only other strong candidate gene based on its immune function in the *Idd18.2* locus is *Nras*. The coding sequence for *Nras* was sequenced previously (4), and polymorphisms between NOD and B6 were not identified. The NGS data show that there is little variation between NOD/ShiLtJ and B6 around *Nras* (Fig. 2A), and the only polymorphism detected in *Nras* is an ambiguous SNP call [C/Y]. The single polymorphism in *Nras* is highly unlikely to be functional because it is located in the polypyrimidine track of intron 4, and both NOD- and B6-derived alleles encode for pyrimidines.

### The function and association of the CD2/CD48/CD58 pathway with several autoimmune diseases highlights *Cd2* as a strong functional candidate for *Idd18.4*

CD2 is a cell-surface molecule expressed on T cells, NK cells, B cells, dendritic cells, and monocytes (56–58). In humans, the primary ligand of CD2 is CD58. However, in mice and rats, a CD58 ortholog has not been identified, and CD48 is believed to be the primary ligand because it is observed to bind to CD2 with much higher affinity in rodents than in humans (16). CD2 and CD58/CD48 interactions have an important role in the formation of the immunologic synapse. Clustering of CD2-CD58/CD48 molecules has been implicated in the repositioning of cell surface molecules to the immunologic synapse and is believed to position T cell and APC membranes at a distance that is suitable for TCR:peptide-MHC complex association (17, 59, 60). Blocking the interaction of CD2 and CD58 by mAbs inhibits T cell proliferation, T and NK cell cytolysis, and production of cytokines by T cells (61), confirming the critical role this ligand pair has in immune signaling and the functional candidacy of CD2 for the *Idd18.4* locus. Moreover, the CD2/CD58 pathway is associated with the human autoimmune diseases, multiple sclerosis, and rheumatoid arthritis (18, 19), and the CD2/CD48 pathway is associated with a model of murine lupus (20). The CD58 protective allele has higher mRNA expression in lymphoblastic cell lines and mononuclear cells from multiple sclerosis patients than the susceptible allele does (18). Giving additional support to the protective effect of higher CD58 expression is the observation that patients with active disease have lower expression of CD58 transcript in cerebrospinal fluid (62), whereas patients in remission have higher levels of CD58 mRNA expression (18). In our studies, we identified that the B6-derived protective alleles of the *Idd18.4* locus result in 1.5-fold higher expression of CD2 protein. These alleles are analogous to the protective allele in human multiple sclerosis, suggesting that higher levels of CD2/CD58 expression and function have a protective effect against autoimmunity.

### The potential interactions of the *Idd10*, *Idd18.2*, and *Idd18.1* candidate genes and the identification of future T1D candidate genes

As we have strong functional candidates for the *Idd10*, *Idd18.2*, and *Idd18.1* loci, it is interesting to speculate how the immunologic roles of these candidates could result in the ability of the B6-derived *Idd18.2* alleles to mask the protection associated with *Idd10* or other subregions of *Idd18*. Although, we are not sure whether *Idd18.3*, *Idd18.1*, or a combination of both are providing the protection observed in line 3539, we have a firm functional candidate gene (*Vav3)* for *Idd18.1* only. *Vav3* encodes a guanine nucleotide exchange factor that is important in the signaling cascade leading to cytoskeletal plasticity, and we have previously observed that protective B6-derived alleles of *Vav3* have ∼50% lower mRNA expression in thymocytes compared with susceptible NOD-derived alleles (7). If present in the same cell type as PEP, VAV3 protein would function downstream of PEP in the immune receptor signaling cascade. It could be that B6-derived susceptible alleles encoding PEP strongly inhibit immune signaling, resulting in reduced signal transduction through the VAV3 pathway, and subsequently the observation that the protection associated with the reduced expression of *Vav3* is not observed. The *Idd10* candidate, *Cd101*, encodes a transmembrane protein expressed on several immune cells and has been suggested to downregulate T cell activation (22, 44). In this scenario, we would expect CD101 to function on a branch of the immune pathway that is upstream of PEP. Susceptible B6-derived PEP may downregulate immune signaling much more strongly than CD101 and, therefore, the effect of variations in CD101 are negligible compared with the susceptible PEP-induced inhibition of immune signaling. Additional experiments are required to investigate these hypotheses concerning interacting molecular and cellular pathways influenced by *Idd* genes.

## Supplementary Material

Data Supplement

## References

[1] Kikutani H. Makino S. 1992 The murine autoimmune diabetes model: NOD and related strains. Adv. Immunol. 51: 285–322. 1323922 10.1016/s0065-2776(08)60490-3

[2] Driver J. P. Serreze D. V. Chen Y. G. 2011 Mouse models for the study of autoimmune type 1 diabetes: a NOD to similarities and differences to human disease. Semin. Immunopathol. 33: 67–87. 20424843 10.1007/s00281-010-0204-1

[3] Ridgway W. M. Peterson L. B. Todd J. A. Rainbow D. B. Healy B. Burren O. S. Wicker L. S. 2008 Gene-gene interactions in the NOD mouse model of type 1 diabetes. Adv. Immunol. 100: 151–175. 19111166 10.1016/S0065-2776(08)00806-7

[4] Lyons P. A. Armitage N. Lord C. J. Phillips M. S. Todd J. A. Peterson L. B. Wicker L. S. 2001 Mapping by genetic interaction: high-resolution congenic mapping of the type 1 diabetes loci *Idd10* and *Idd18* in the NOD mouse. Diabetes 50: 2633–2637. 11679445 10.2337/diabetes.50.11.2633

[5] Podolin P. L. Denny P. Armitage N. Lord C. J. Hill N. J. Levy E. R. Peterson L. B. Todd J. A. Wicker L. S. Lyons P. A. 1998 Localization of two insulin-dependent diabetes (*Idd*) genes to the *Idd10* region on mouse chromosome 3. Mamm. Genome 9: 283–286. 9530623 10.1007/s003359900749

[6] Podolin P. L. Denny P. Lord C. J. Hill N. J. Todd J. A. Peterson L. B. Wicker L. S. Lyons P. A. 1997 Congenic mapping of the insulin-dependent diabetes (*Idd*) gene, *Idd10*, localizes two genes mediating the *Idd10* effect and eliminates the candidate *Fcgr1.* J. Immunol. 159: 1835–1843. 9257847

[7] Fraser H. I. Dendrou C. A. Healy B. Rainbow D. B. Howlett S. Smink L. J. Gregory S. Steward C. A. Todd J. A. Peterson L. B. Wicker L. S. 2010 Nonobese diabetic congenic strain analysis of autoimmune diabetes reveals genetic complexity of the *Idd18* locus and identifies *Vav3* as a candidate gene. J. Immunol. 184: 5075–5084. 20363978 10.4049/jimmunol.0903734 PMC2886967

[8] Ghosh S. Palmer S. M. Rodrigues N. R. Cordell H. J. Hearne C. M. Cornall R. J. Prins J. B. McShane P. Lathrop G. M. Peterson L. B. 1993 Polygenic control of autoimmune diabetes in nonobese diabetic mice. Nat. Genet. 4: 404–409. 8401590 10.1038/ng0893-404

[9] Brodnicki T. C. Quirk F. Morahan G. 2003 A susceptibility allele from a non-diabetes-prone mouse strain accelerates diabetes in NOD congenic mice. Diabetes 52: 218–222. 12502517 10.2337/diabetes.52.1.218

[10] Hunter K. Rainbow D. Plagnol V. Todd J. A. Peterson L. B. Wicker L. S. 2007 Interactions between *Idd5.1*/*Ctla4* and other type 1 diabetes genes. J. Immunol. 179: 8341–8349. 18056379 10.4049/jimmunol.179.12.8341

[11] Stanford S. M. Bottini N. 2014 PTPN22: the archetypal non-HLA autoimmunity gene. Nat. Rev. Rheumatol. 10: 602–611. 25003765 10.1038/nrrheum.2014.109 PMC4375551

[12] Sarmiento J. Wallis R. H. Ning T. Marandi L. Chao G. Veillette A. Lernmark Å. Paterson A. D. Poussier P. 2015 A functional polymorphism of Ptpn22 is associated with type 1 diabetes in the BioBreeding rat. J. Immunol. 194: 615–629. 25505293 10.4049/jimmunol.1302689

[13] Hill R. J. Zozulya S. Lu Y. L. Ward K. Gishizky M. Jallal B. 2002 The lymphoid protein tyrosine phosphatase Lyp interacts with the adaptor molecule Grb2 and functions as a negative regulator of T-cell activation. Exp. Hematol. 30: 237–244. 11882361 10.1016/s0301-472x(01)00794-9

[14] Hasegawa K. Yajima H. Katagiri T. Ogimoto M. Arimura Y. Mitomo K. Mashima K. Mizuno K. Yakura H. 1999 Requirement of PEST domain tyrosine phosphatase PEP in B cell antigen receptor-induced growth arrest and apoptosis. Eur. J. Immunol. 29: 887–896. 10092092 10.1002/(SICI)1521-4141(199903)29:03<887::AID-IMMU887>3.0.CO;2-9

[15] Arechiga A. F. Habib T. He Y. Zhang X. Zhang Z. Y. Funk A. Buckner J. H. 2009 Cutting edge: the PTPN22 allelic variant associated with autoimmunity impairs B cell signaling. J. Immunol. 182: 3343–3347. 19265110 10.4049/jimmunol.0713370 PMC2797545

[16] Brown M. H. Boles K. van der Merwe P. A. Kumar V. Mathew P. A. Barclay A. N. 1998 2B4, the natural killer and T cell immunoglobulin superfamily surface protein, is a ligand for CD48. J. Exp. Med. 188: 2083–2090. 9841922 10.1084/jem.188.11.2083 PMC2212392

[17] Zaru R. Cameron T. O. Stern L. J. Müller S. Valitutti S. 2002 Cutting edge: TCR engagement and triggering in the absence of large-scale molecular segregation at the T cell-APC contact site. J. Immunol. 168: 4287–4291. 11970969 10.4049/jimmunol.168.9.4287

[18] De Jager P. L. Baecher-Allan C. Maier L. M. Arthur A. T. Ottoboni L. Barcellos L. McCauley J. L. Sawcer S. Goris A. Saarela J. 2009 The role of the *CD58* locus in multiple sclerosis. Proc. Natl. Acad. Sci. USA 106: 5264–5269. 19237575 10.1073/pnas.0813310106 PMC2664005

[19] Raychaudhuri S. Thomson B. P. Remmers E. F. Eyre S. Hinks A. Guiducci C. Catanese J. J. Xie G. Stahl E. A. Chen R. 2009 Genetic variants at *CD28, PRDM1* and *CD2*/*CD58* are associated with rheumatoid arthritis risk. Nat. Genet. 41: 1313–1318. 19898481 10.1038/ng.479 PMC3142887

[20] Wandstrat A. E. Nguyen C. Limaye N. Chan A. Y. Subramanian S. Tian X. H. Yim Y. S. Pertsemlidis A. Garner H. R. Jr. Morel L. Wakeland E. K. 2004 Association of extensive polymorphisms in the SLAM/CD2 gene cluster with murine lupus. Immunity 21: 769–780. 15589166 10.1016/j.immuni.2004.10.009

[21] Rozen S. Skaletsky H. 2000 Primer3 on the WWW for general users and for biologist programmers. Methods Mol. Biol. 132: 365–386. 10547847 10.1385/1-59259-192-2:365

[22] Rainbow D. B. Moule C. Fraser H. I. Clark J. Howlett S. K. Burren O. Christensen M. Moody V. Steward C. A. Mohammed J. P. 2011 Evidence that *Cd101* is an autoimmune diabetes gene in nonobese diabetic mice. J. Immunol. 187: 325–336. 21613616 10.4049/jimmunol.1003523 PMC3128927

[23] Mott R. 1997 EST_GENOME: a program to align spliced DNA sequences to unspliced genomic DNA. Comput. Appl. Biosci. 13: 477–478. 9283765 10.1093/bioinformatics/13.4.477

[24] Steward C. A. Humphray S. Plumb B. Jones M. C. Quail M. A. Rice S. Cox T. Davies R. Bonfield J. Keane T. M. 2010 Genome-wide end-sequenced BAC resources for the NOD/MrkTac() and NOD/ShiLtJ() mouse genomes. Genomics 95: 105–110. 19909804 10.1016/j.ygeno.2009.10.004 PMC2824108

[25] Ning Z. Cox A. J. Mullikin J. C. 2001 SSAHA: a fast search method for large DNA databases. Genome Res. 11: 1725–1729. 11591649 10.1101/gr.194201 PMC311141

[26] Smit, A. F. A., R. Hubley, and P. Green. *RepeatMasker Open-4.0*. 2013–2015. Available at: http://www.repeatmasker.org.

[27] Pruitt K. D. Tatusova T. Klimke W. Maglott D. R. 2009 NCBI Reference Sequences: current status, policy and new initiatives. Nucleic Acids Res. 37: D32–D36. 18927115 10.1093/nar/gkn721 PMC2686572

[28] Pruitt K. D. Harrow J. Harte R. A. Wallin C. Diekhans M. Maglott D. R. Searle S. Farrell C. M. Loveland J. E. Ruef B. J. 2009 The consensus coding sequence (CCDS) project: Identifying a common protein-coding gene set for the human and mouse genomes. Genome Res. 19: 1316–1323. 19498102 10.1101/gr.080531.108 PMC2704439

[29] Meyer L. R. Zweig A. S. Hinrichs A. S. Karolchik D. Kuhn R. M. Wong M. Sloan C. A. Rosenbloom K. R. Roe G. Rhead B. 2013 The UCSC Genome Browser database: extensions and updates 2013. Nucleic Acids Res. 41: D64–D69. 23155063 10.1093/nar/gks1048 PMC3531082

[30] Flicek P. Ahmed I. Amode M. R. Barrell D. Beal K. Brent S. Carvalho-Silva D. Clapham P. Coates G. Fairley S. 2013 Ensembl 2013. Nucleic Acids Res. 41: D48–D55. 23203987 10.1093/nar/gks1236 PMC3531136

[31] Hulbert E. M. Smink L. J. Adlem E. C. Allen J. E. Burdick D. B. Burren O. S. Cassen V. M. Cavnor C. C. Dolman G. E. Flamez D. 2007 T1DBase: integration and presentation of complex data for type 1 diabetes research. Nucleic Acids Res. 35: D742–D746. 17169983 10.1093/nar/gkl933 PMC1781218

[32] Stein L. D. Mungall C. Shu S. Caudy M. Mangone M. Day A. Nickerson E. Stajich J. E. Harris T. W. Arva A. Lewis S. 2002 The generic genome browser: a building block for a model organism system database. Genome Res. 12: 1599–1610. 12368253 10.1101/gr.403602 PMC187535

[33] Altschul S. F. Gish W. Miller W. Myers E. W. Lipman D. J. 1990 Basic local alignment search tool. J. Mol. Biol. 215: 403–410. 2231712 10.1016/S0022-2836(05)80360-2

[34] Schneider C. A. Rasband W. S. Eliceiri K. W. 2012 NIH Image to ImageJ: 25 years of image analysis. Nat. Methods 9: 671–675. 22930834 10.1038/nmeth.2089 PMC5554542

[35] Bottini N. Peterson E. J. 2014 Tyrosine phosphatase PTPN22: multifunctional regulator of immune signaling, development, and disease. Annu. Rev. Immunol. 32: 83–119. 24364806 10.1146/annurev-immunol-032713-120249 PMC6402334

[36] Burset M. Seledtsov I. A. Solovyev V. V. 2000 Analysis of canonical and non-canonical splice sites in mammalian genomes. Nucleic Acids Res. 28: 4364–4375. 11058137 10.1093/nar/28.21.4364 PMC113136

[37] Liu Y. Stanford S. M. Jog S. P. Fiorillo E. Orrú V. Comai L. Bottini N. 2009 Regulation of lymphoid tyrosine phosphatase activity: inhibition of the catalytic domain by the proximal interdomain. Biochemistry 48: 7525–7532. 19586056 10.1021/bi900332f PMC3113683

[38] Coolidge C. J. Seely R. J. Patton J. G. 1997 Functional analysis of the polypyrimidine tract in pre-mRNA splicing. Nucleic Acids Res. 25: 888–896. 9016643 10.1093/nar/25.4.888 PMC146492

[39] Janssen R. J. Wevers R. A. Häussler M. Luyten J. A. Steenbergen-Spanjers G. C. Hoffmann G. F. Nagatsu T. Van den Heuvel L. P. 2000 A branch site mutation leading to aberrant splicing of the human tyrosine hydroxylase gene in a child with a severe extrapyramidal movement disorder. Ann. Hum. Genet. 64: 375–382. 11281275 10.1046/j.1469-1809.2000.6450375.x

[40] Zarudnaya M. I. Kolomiets I. M. Potyahaylo A. L. Hovorun D. M. 2003 Downstream elements of mammalian pre-mRNA polyadenylation signals: primary, secondary and higher-order structures. Nucleic Acids Res. 31: 1375–1386. 12595544 10.1093/nar/gkg241 PMC149834

[41] Thanaraj T. A. Clark F. 2001 Human GC-AG alternative intron isoforms with weak donor sites show enhanced consensus at acceptor exon positions. Nucleic Acids Res. 29: 2581–2593. 11410667 10.1093/nar/29.12.2581 PMC55748

[42] Cloutier J. F. Veillette A. 1999 Cooperative inhibition of T-cell antigen receptor signaling by a complex between a kinase and a phosphatase. J. Exp. Med. 189: 111–121. 9874568 10.1084/jem.189.1.111 PMC1887684

[43] Hasegawa K. Martin F. Huang G. Tumas D. Diehl L. Chan A. C. 2004 PEST domain-enriched tyrosine phosphatase (PEP) regulation of effector/memory T cells. Science 303: 685–689. 14752163 10.1126/science.1092138

[44] Penha-Gonçalves C. Moule C. Smink L. J. Howson J. Gregory S. Rogers J. Lyons P. A. Suttie J. J. Lord C. J. Peterson L. B. 2003 Identification of a structurally distinct CD101 molecule encoded in the 950-kb *Idd10* region of NOD mice. Diabetes 52: 1551–1556. 12765969 10.2337/diabetes.52.6.1551

[45] Paigen K. Szatkiewicz J. P. Sawyer K. Leahy N. Parvanov E. D. Ng S. H. Graber J. H. Broman K. W. Petkov P. M. 2008 The recombinational anatomy of a mouse chromosome. PLoS Genet. 4: e1000119. 18617997 10.1371/journal.pgen.1000119 PMC2440539

[46] Yamanouchi J. Rainbow D. Serra P. Howlett S. Hunter K. Garner V. E. Gonzalez-Munoz A. Clark J. Veijola R. Cubbon R. 2007 Interleukin-2 gene variation impairs regulatory T cell function and causes autoimmunity. Nat. Genet. 39: 329–337. 17277778 10.1038/ng1958 PMC2886969

[47] Cordell H. J. Todd J. A. Hill N. J. Lord C. J. Lyons P. A. Peterson L. B. Wicker L. S. Clayton D. G. 2001 Statistical modeling of interlocus interactions in a complex disease: rejection of the multiplicative model of epistasis in type 1 diabetes. Genetics 158: 357–367. 11333244 10.1093/genetics/158.1.357 PMC1461617

[48] Wu J. Katrekar A. Honigberg L. A. Smith A. M. Conn M. T. Tang J. Jeffery D. Mortara K. Sampang J. Williams S. R. 2006 Identification of substrates of human protein-tyrosine phosphatase PTPN22. J. Biol. Chem. 281: 11002–11010. 16461343 10.1074/jbc.M600498200

[49] Fiorillo E. Orrú V. Stanford S. M. Liu Y. Salek M. Rapini N. Schenone A. D. Saccucci P. Delogu L. G. Angelini F. 2010 Autoimmune-associated PTPN22 R620W variation reduces phosphorylation of lymphoid phosphatase on an inhibitory tyrosine residue. J. Biol. Chem. 285: 26506–26518. 20538612 10.1074/jbc.M110.111104 PMC2924087

[50] Zikherman J. Hermiston M. Steiner D. Hasegawa K. Chan A. Weiss A. 2009 *PTPN22* deficiency cooperates with the CD45 E613R allele to break tolerance on a non-autoimmune background. J. Immunol. 182: 4093–4106. 19299707 10.4049/jimmunol.0803317 PMC2765978

[51] Salmond R. J. Brownlie R. J. Morrison V. L. Zamoyska R. 2014 The tyrosine phosphatase PTPN22 discriminates weak self peptides from strong agonist TCR signals. Nat. Immunol. 15: 875–883. 25108421 10.1038/ni.2958 PMC4148831

[52] Maine C. J. Hamilton-Williams E. E. Cheung J. Stanford S. M. Bottini N. Wicker L. S. Sherman L. A. 2012 PTPN22 alters the development of regulatory T cells in the thymus. J. Immunol. 188: 5267–5275. 22539785 10.4049/jimmunol.1200150 PMC3358490

[53] Zheng P. Kissler S. 2013 *PTPN22* silencing in the NOD model indicates the type 1 diabetes-associated allele is not a loss-of-function variant. Diabetes 62: 896–904. 23193190 10.2337/db12-0929 PMC3581188

[54] Brownlie R. J. Miosge L. A. Vassilakos D. Svensson L. M. Cope A. Zamoyska R. 2012 Lack of the phosphatase PTPN22 increases adhesion of murine regulatory T cells to improve their immunosuppressive function. Sci. Signal. 5: ra87. 23193160 10.1126/scisignal.2003365 PMC5836999

[55] Maine C. J. Marquardt K. Cheung J. Sherman L. A. 2014 PTPN22 controls the germinal center by influencing the numbers and activity of T follicular helper cells. J. Immunol. 192: 1415–1424. 24453256 10.4049/jimmunol.1302418 PMC3933017

[56] Bierer B. E. Sleckman B. P. Ratnofsky S. E. Burakoff S. J. 1989 The biologic roles of CD2, CD4, and CD8 in T-cell activation. Annu. Rev. Immunol. 7: 579–599. 2653377 10.1146/annurev.iy.07.040189.003051

[57] Crawford K. Stark A. Kitchens B. Sternheim K. Pantazopoulos V. Triantafellow E. Wang Z. Vasir B. Larsen C. E. Gabuzda D. 2003 CD2 engagement induces dendritic cell activation: implications for immune surveillance and T-cell activation. Blood 102: 1745–1752. 12714509 10.1182/blood-2002-07-2206

[58] Kingma D. W. Imus P. Xie X. Y. Jasper G. Sorbara L. Stewart C. Stetler-Stevenson M. 2002 CD2 is expressed by a subpopulation of normal B cells and is frequently present in mature B-cell neoplasms. Cytometry 50: 243–248. 12360573 10.1002/cyto.10131

[59] Dustin M. L. Golan D. E. Zhu D. M. Miller J. M. Meier W. Davies E. A. van der Merwe P. A. 1997 Low affinity interaction of human or rat T cell adhesion molecule CD2 with its ligand aligns adhering membranes to achieve high physiological affinity. J. Biol. Chem. 272: 30889–30898. 9388235 10.1074/jbc.272.49.30889

[60] van der Merwe P. A. McNamee P. N. Davies E. A. Barclay A. N. Davis S. J. 1995 Topology of the CD2-CD48 cell-adhesion molecule complex: implications for antigen recognition by T cells. Curr. Biol. 5: 74–84. 7697352 10.1016/s0960-9822(95)00019-4

[61] Springer T. A. Dustin M. L. Kishimoto T. K. Marlin S. D. 1987 The lymphocyte function-associated LFA-1, CD2, and LFA-3 molecules: cell adhesion receptors of the immune system. Annu. Rev. Immunol. 5: 223–252. 3109455 10.1146/annurev.iy.05.040187.001255

[62] Brynedal, B., I. L. Bomfim, T. Olsson, K. Duvefelt, and J. Hillert. 2009. Differential expression, and genetic association, of CD58 in Swedish multiple sclerosis patients. Proc. Natl. Acad. Sci. U. S. A. 106: E58, author reply E59. 10.1073/pnas.0904338106 PMC2695099 19497873

