# Supplementary material for: Ptpn22 and Cd2 Variations Are Associated with Altered Protein Expression and Susceptibility to Type 1 Diabetes in Nonobese Diabetic Mice
Source: J Immunol. 2015 Oct 5;195(10):4841–52. doi: 10.4049/jimmunol.1402654 (PMC4635565; doi:10.4049/jimmunol.1402654)
Supplement: Data Supplement [file JI_1402654.zip › JI_1402654_Supplemental_Material_1.pdf]

Supplemental Table I. Novel microsatellite markers and RFLPs designed to define the boundaries of the *Idd18.2* and *Idd18.4* loci and other *Idd* loci on chromosome 3.

Marker	Forward Primer	Reverse Primer	Product Length (bp) NOD	B6	Type of marker
Ptgfrn_Int1_SNP_2	GGGACGCTCGTCTATCCTTT	GGCTCACTTTTCCTTGAAGC	292	292	Sequencing [NOD = G, B6 = T]
AL672281_7	GAACCAAGGCAGGCATTAAA	TTACTGCCCCCTTCTCCTTT	212	210	Microsatellite
2410_Micro_1	AGGAGAATCTTCTCAGAGCCTTA	TGAATCTTTATAGACATGTGATG GA	111	131	Microsatellite
2410_p_SNP_5	AGCAAGATCTTTAGCTGTTGTTAAT	AGTTTGCTGTCTTGGGACAT	116 + 98	214	RFLP with AvaI
Chr3-101-864	TCACAGCCACACCCTGATAG	AAGGGTCATGGGGCTTTTAT	129	53 + 76	RFLP with NlaIV
AC122219_3	ACCCACTCACTAACTCACATAGG	ACACACACACAGTGGATGGA	293	311	Microsatellite
R8_p_SNP_1	CCTGCATAGTTGGTTCAACAC	TGCTCCTTAGACAGGCTCTTC	100 + 101	201	RFLP with AciI
Magi3	GGGATGAGACCACTGAGAAGC	TGTACGTATGTTTCCAGAGCTGA	136	146	Microsatellite
Susc_96.62	AGCCAGGACTGTAGTGGTGT	TTGCTCTTGGTCATAGTGCTT	178	208	Microsatellite
R8_micro_1	GAAGACCATCAGCAGCAACT	AGCCTATGCTGGGACCTCT	198	196	Microsatellite
R8_micro_2	CGAGTTGGGAGCATAGTCAA	ATCTGGGATTCTGTGCTGAG	190	188	Microsatellite
AC093365_1	CTAATGGGGTAGGCTGACCA	TTCTCCCAGACTCATGGCTT	262	259	Microsatellite
AC093365_6	ACTGCTGCTTTTGATCCCTG	AGGTAGCCAGATCTTGGGGT	271	273	Microsatellite
RS38124092	GCCCATATGATCCAATCACC	ATGGGTGGCATTATGGCTTA	91 + 24	115	RFLP with RsaI

Supplemental Table IIA. SNPs in coding sequence of *Ptpn22*.

SNP[1] [B6/NOD]	Exon[2]	Synonymous (residue number, amino acid)	dbSNP in same position	Sequence flanking SNP [B6/NOD]
T/C (90937)	Exon 4 (90860-90955)	Yes (117, I)	rs30006107	CTGGACTTCTGGAGGATGAT[T/C]TGGGAGTACCGCATCTTGGT
A/G (92439)	Exon 6 (92413-92484)	Yes (145, E)	rs6384342	TGTGAGCGTTATTGGGCCGA[A/G]CCAGGAGAAACGCAGCTGCA
A/G (102031)	Exon 12 (102002-102078)	Yes (315, Q)	none	CAATGCTCAATTCCTGAACA[A/G]AGCCTCACGGTAGAAGCTGA
G/A (102041)	Exon 12 (102002-102078)	No (319, V to I)	rs33557973	TTCCTGAACAAAGCCTCACG[G/A]TAGAAGCTGACTCTTGTCCT
T/C (102474)	Exon 13 (102207-103019)	Yes (420, F)	rs33559904	AAGTATCAAAGTCTGGATTT[T/C]GGTTCCATGTTGTTTGGGTC
G/A (102852)	Exon 13 (102207-103019)	Yes (546, S)	none	CCTCCGAATAGTGCTGATTC[G/A]AAGATGTCTTTTGATCTGCC

[1,2] The position of the SNPs and exons within the B6 clone AC124698.4 is shown in parenthesis.

Supplemental Table IIB. ESTs representing alternatively spliced *Ptpn22* transcripts and RT-PCR primers to confirm existence.

Identifying ESTs	Alternative transcript[1,2]	Description	Exons present	Forward primer<br>Reverse primer
CF911307.1<br>CF913254.1<br>CF911839.1	*Ptpn22_Ex_7a*	cryptic 3' splice acceptor in exon 7, causes 4bp deletion in exon 7 (exon 7a) and exon 8 spliced out	2, 3, 4, 5, 6, 7a, 9, 10	For-CATTGTCATGGCATGTATGGAG<br>Rev-TTCTCAGACAGGATATAGAA
AA445561	*Ptpn22_delta_16*	Exon 16 spliced out	13, 14, 15, 17, 18	For-TTGTGGTTGAGGAAGCCG<br>Rev-TGATGTCGATCTTGCTTGGTG
CK790128.1	*Ptpn22_Ex2_novel5utr*<br>***(Ptpn22_F)***	Novel 5' UTR between exons 1 and 2 (exon 1a)	1a	For-GTAGGGATAACACGACCATG<br>Rev-CTCTGAGCCACAGTTGTAGG
BM245011.2<br>BE447740.1	*Ptpn22_Ex17_novel5utr*<br>***(Ptpn22_E)***	Novel 5' UTR immediately upstream of exon 17 (exon 17a)	17a, 18, 19, 20, 21	For-AGGTTGCAGGCCAAAAAG<br>Rev-AAATTCAGAAAGGAGCTGGAA
CA574365<br>CA574366<br>BM241317<br>BM241318	*Ptpn22_Ex17_4bpDel*<br>***(Ptpn22_D)***	4 bp deletion in middle of exon 17 (Exon 17b)	17b, 18, 19, 20, 21	For-TATTGTGGTTGAGGAAGCCG<br>Rev-TATTGTGGTTGAGGAAGCCG
BY747853<br>BB638938	*Ptpn22_Ex_1_ext*	Run through into intron 1 (exon 1b)	1b	For-AACCTACTCACAGACTCTTG<br>Rev-CATGGTCGTGTTATCCCTAC

[1] All transcripts except for Ptpn22_delta_16 and *Ptpn22_Ex_7a* were detected by RT-PCR in the spleen and/or kidney samples tested.
[2] The names of the qPCR primer/probe sets used to detect transcripts are given in parentheses.

Supplemental Table IIC. *Ptpn22* alternatively spliced transcripts detected by RT-PCR.

Alternative transcript[1]	Description	Exons present
Ptpn22_all_intron_14 ***(Ptpn22_H)***	All of intron 14 included (exon 14a)	13, 14a, 15
Ptpn22_delta_12_13_plus_in_14 ***(Ptpn22_G)***	Exons 12, 13 spliced out and all of intron 14 included (exon 14a)	4 to 11, 14a, 15, 16, 17
Ptpn22_delta_15 ***(Ptpn22_K)***	Exon 15 spliced out	2 to 14, 16 to 21
Ptpn22_Ex_1_17	Exon 2 to 16 spliced out	1, 17 to 21
Ptpn22_Ex_1_20	Exon 2 to 19	1, 20, 21
Ptpn22_Ex_1_21	Exon 2 to 20	1, 21
Ptpn22_delta_12_13_14_plus_in_14	Exons 12, 13, and 14 spliced out and half of intron 14 included (exon 14b)	1 to 11, 14b to 20
Ptpn22_delta_12_13 ***(Ptpn22_I)***	Exons 12, 13 spliced out	1 to 11, 14 to 21
Ptpn22_delta_12_13_plus14bp_in14 ***(Ptpn22_J)***	Exons 12, 13 spliced out, 14 bp of start of intron 14 included (exon 14c)	1 to 11, 14c to 21

[1] The names of the qPCR primer/probe sets used to detect transcripts are given in parentheses.

Supplemental Table IID. Primer and probe sets for analyzing full-length and *Ptpn22* alternatively spliced transcripts by qPCR.

Transcript	Forward primer	Reverse primer	Probe
Full-length *Ptpn22*	GGTTGAGGAAGCCGGAGAG	TCAGATGTCCCACTGCATTC	CCCTCACCACGTGTTACCGAATCC
*Ptpn22*_D	TCGACATCAGGATGGTTCTC	CCTCATCAGCAAGAAGGACTC	TCCACCTCCTCTCCCAGAAAGAACTC
*Ptpn22*_E	TCTATTTTACAGATCGACATCAGGA	CCTCATCAGCAAGAAAGAAGG	TCTCCTCCACCTCCTCTCCCAGAA
*Ptpn22*_F	GGTTTTCTCCAGAAGCTGAAA	GGCCTCTGAGCCACAGTT	CAATCCACCAAGTACAAGGCCGACA
*Ptpn22*_G	CACCTTGGAAGAGAGCTCCTT	GACTGTACTCACCGGCTTCC	CCGCCACTCCCTGAACGGAC
*Ptpn22*_H	AGCTGTAGAAGCTCCTTCTCG	GACTGTACTCACCGGCTTCC	CCGCCACTCCCTGAACGGAC
*Ptpn22*_I	CACCTTGGAAGAGAGCTCCTT	GGCTCTCCGGCTTCCT	CCGCCACTCCCTGAACGGAC
*Ptpn22*_J	CACCTTGGAAGAGAGCTCCTT	TGAGGGCTCTCTGACTGTACTC	CCGCCACTCCCTGAACGGAC
*Ptpn22*_K	AGCTGTAGAAGCTCCTTCTCG	GGAGTTTCACATTCGGCTTC	CCGCCACTCCCTGAACGGAC
β2-microglobulin	GCTATCCAGAAAACCCCTCAAAT	CTGTGTTACGTAGCAGTTCAGTATGTTC	AGTATACTCACGCCACCCACCGGAGAAT

Supplemental Table III. Polymorphisms between NOD and B6 in *Cd2*.

Polymorphism[1,2]	Region of *Cd2*[3]	dbSNP in same position	Sequence flanking polymorphism [B6/NOD]
(GTGT/- 78705) INDEL in GT microsatellite	Upstream of start of 5' UTR (80689)	n/a	TTGTCGGTAATTTGCCTTTTTTATTACCTAGCTTTTCAATTTTTTAATAT[GTGT/-] GTGTGTGTGTGTGTGTGTGTGTGTGTGTGTATGTATATATATATATTACC
(-/AT 78742) INDEL in AT microsatellite	Upstream of start of 5' UTR (80689)	n/a	AATTTTTTAATATGTGTGTGTGTGTGTGTGTGTGTGTGTGTGTGTGTATG[-/TA] TATATATATATATTACCTAGGAAAGTTTTACGTCCTACAGAGTCAGACCT
(AG/- 80188) INDEL in AG microsatellite	Upstream of start of 5' UTR (80689)	n/a	ATCCACAGCAGGAGACAGACAGATAGACAGAGTGGGGAAGGGGAGAGGGA[AG/-] AGAGAGAGAGAGAGAGAGAGAGAGCTAACAGGACCACAAGGGCTTTAAAG
(TC/- 80574) INDEL in TC microsatellite	Upstream of start of 5' UTR (80689)	n/a	TTCGGTTAAGGAGGGCAGCAAATGCATGAGTCGTTTTGATAGGGTCTCTG[TC/-] TCTCTCTCTCCTTCCCCATCTCTACCTCTCCCTCTCCCCCTCCCCCTC
(T/C 81595)	Intron 2 (81222-85366)	rs33435432	TTGTCTGGAGAGCTATGTTTAAGCCAGGGTATGATGAGAGGGCATGATAC[T/C] GAAGAGCAACAGGAGAAGACCAACCAGGAGTCCTCTCCTGTCATCCAAGC
(A/G 81877)	Intron 2 (81222-85366)	none	AGATGAGGAAGGGTGCAAACATGCCGGGTGTGGGGGTGAGGAGGAAGTGA[A/G] GAAGCTTGGGTGGGTGAGAAAGAAGAAAACGAAGCCTGGGATGCTGTCGA
(G/T 81942)	Intron 2 (81222-85366)	none	TGAGAAAGAAGAAAACGAAGCCTGGGATGCTGTCGAAGCTGAAAGTCCAG[G/T] GACAAACTGGCAGCTCACACCAATGGGTGAGAGGAGAAAGCCTGCCCTTG
(A/C 82577)	Intron 2 (81222-85366)	rs31137972	ATACAATTCACATACCACACAGTTTGCCCGTTTGCCGTGCGCAGTTCAAC[A/C] GCTTTCTGCATAGTCACAGAGTTACGCGGTTACCACCATAGGCAACATTT
(-/TG 82890) INDEL in TG microsatellite	Intron 2 (81222-85366)	n/a	AGTTTGAGATTAGAAGTGTTTAGGATTGGGGCTTTTGGAAGATTTCAAAA[-/TG] TGTGTGTGTGTGTGTGTGTGTGTGTGTGTGTGTTCTTGGAGATGGGAC
(-/C 84523) INDEL in poly C repeat	Intron 2 (81222-85366)	n/a	GAAATTAACTCAGAACCTTGGGGCTAGGAAGGGCAGCGTCCTTTCTGGCT[-/C] CCCCCCCGCCCCCAACCCCCACGTGAAAACGCAGGAGGGCAGAGATGCTG
(-/TGTGTG 86674) INDEL in TG microsatellite	Intron 3 (85598-88717)	n/a	AGATAAGAGTGAAGCTCACATGTGCTTGGAGGGATCAGACAGCAGTGAAA[-/TGTGTG] TGTGTGTGTGTGTGTGTGTGTGTGTGTGTGTGTGGCTGAAGGCACAGA
(CCC/- 86747) INDEL in poly C repeat	Intron 3 (85598-88717)	n/a	GTGTGTGTGTGTGGCTGAAGGCACAGATGCAGAAGGATTTTATTAACACA[CCC/-] CCCCCCCCCCAGGTTCATGGATGGTCTCCCTCTTCTGCCTTCCTGTCCCC
(AA/- 86862) INDEL in poly A repeat	Intron 3 (85598-88717)	n/a	CCAATCACAAAGGCACGAGGGGCAGGGCAAAGCTGCCTCTCCAAGACTCC[AA/-] AAAAAAAAAAAAAAAAAACAAAAACGTTTTCCTCTTTCTGAAGGCCCTGA
(GAGAGA/- 92138) INDEL in GA microsatellite	Intron 4 (88841-92354)	n/a	AAGAGAGAGCAAGAGAGAGAGAGAGAGAGAGAGAGAGAGAGAGAGAGAGA[GAGAGA/-] ATTAATCTCAAAAAGAAGAAAAGGCGACAGTGCCTAAGAAATGGCATCAG

[1]For all polymorphisms the B6 allele is stated first.
[2]The position of the SNPs or insertion/deletion polymorphism in the B6 clone sequence AC131184.3 is given in parentheses.
[3]The position of the *Cd2* gene regions in the B6 clone sequence AC131184.3 is given in parentheses.

Mus musculus (B6)	HLGREIQAQCSIPEQSLT**V**EADSCPLDLPKNAMRDVKTTNQ
Mus musculus (NOD)	HLGREIQAQCSIPEQSLT**I**EADSCPLDLPKNAMRDVKTTNQ
Rattus norvegicus (EDL85468.1)	HREREIQAECSIPEQKLT**I**EADSYPLDFPKNAVRDAKMTNQ
Homo sapiens (Q9Y2R2.2)	HSGTESQAKHCIPEKNHT**L**QADSYSPNLPKSTTKAAKMMNQ
Pan troglodytes (XP_513663.2)	HSGTESQAKHCIPEKNHT**L**QADSYSPNLPKSTTKAAKMMNQ
Pongo abelii (XP_002810452.1)	HSGTKSQAKYCIPEQNHT**L**QADSYSPNLPKSTIKAAKMMNQ
Equus caballus (XP_001917875.1)	HSGTEIQAKYSLPEQNPT**L**GADSCSPNLSKGSINEAKIKNQ
Oryctolagus cuniculus (XP_002715464.1)	YTGTEVQANYSITEQNPI**L**HAGSHPPNLPKNTIKEARMMKQ
Loxodonta Africana (XP_003409639.1)	YSGTEIQAKYSVPEQNST**L**EADPYPLSLPKSSIKEATMMNQ
Heterocephalus glaber (EHB00296.1)	QSGRKIQAKYSIPEQNPT**P**QADFCSPNLPKGSIKEAKMMNQ
Canis lupus familiaris (XP_540240.3)	YSGTEMQAKYSVLEQNPT**V**ETESYSPNLPESDIKEAKMMNQ
Bos taurus (NP_001179432.2)	HSGTEIQAKDSVFEQNPN**I**EAKSYSPSLPKSNIKEATVMNQ

Supplemental Figure 1. PEP/LYP protein sequence alignment spanning residue 319, which codes for valine in B6 and isoleucine in NOD.

To determine if the non-synonymous SNP (G/A 102041) present between NOD and B6 mice that codes for valine in B6 and isoleucine in NOD at residue 319 could affect the protein function of PEP we produced a multiple species alignment of PEP/LYP in order to see if these residues are evolutionarily conserved, which could suggest a necessary role for protein function. The PEP/LYP structure consists of a conserved phosphatase domain, a non-conserved interdomain, and a conserved PEST-rich region that contains proline-rich regions. A section of the multiple alignment that spans residue 319 (in bold and highlighted green) is shown, this is located in the interdomain region. Identical residues are highlighted in yellow. Overall, this region seems to be unconserved with only 4 residues identical in all species. Residue 319 is not conserved, although most residues are hydrophobic aliphatic except for the proline present in *Heterocephalus glaber*. Based on the location of residue 319 in the interdomain (outside the two known functional domains), the lack of conserved residues surrounding 319, and the fact that both NOD and B6 mice have the conserved hydrophobic aliphatic residue at 319, it is unlikely that this non-synonymous polymorphism affects the function of PEP. However, recent studies have identified that the first 20 amino acids of the interdomain region of LYP (residues 301-320) are involved in inhibiting the activity of the neighboring phosphatase domain due to intramolecular interactions with the phosphatase domain (37). If the first 20 residues of the PEP interdomain (301-320) have the same function as that of LYP, residue 319 is in a location where it could alter the binding efficiency of the interdomain between NOD and B6. However, as the side chains of valine and isoleucine are very similar, and the lack of conservation between PEP and LYP in the interdomain could mean a different region of the interdomain is involved in inhibition of the PEP phosphatase domain, we still believe it unlikely that the non-synonymous SNP between NOD and B6 will alter the activity of PEP.
